# Bronchopulmonary Dysplasia and Innate Immunity: A Narrative Review of the Roles of IL-1β and IL-8 (CXCL8)

**DOI:** 10.3390/children13070888

**Published:** 2026-07-01

**Authors:** Dubravka Bačaj Ivanić, Štefan Grosek, Andreja Nataša Kopitar

**Affiliations:** 1 Department of Gynecology and Obstetrics, Clinical Hospital Sveti Duh, 10000 Zagreb, Croatia; dbacajivanic@kbsd.hr; 2 Department of Perinatology, Division of Obstetrics and Gynecology, University Medical Centre Ljubljana, 1000 Ljubljana, Slovenia; stefan.grosek@kclj.si; 3 Department of Medical Ethics, Faculty of Medicine, University of Ljubljana, 1000 Ljubljana, Slovenia; 4 Institute for Microbiology and Immunology, Faculty of Medicine, University of Ljubljana, 1000 Ljubljana, Slovenia

**Keywords:** premature infant, chronic lung disease, inflammation, cytokines, chemokines

## Abstract

**Highlights:**

**What are the main findings?**

- Human and animal studies consistently demonstrate an association between elevated IL-1β and IL-8 (CXCL8) levels and the development of bronchopulmonary dysplasia in preterm infants.
- Activation of innate immune pathways, particularly IL-1β- and IL-8-mediated inflammatory responses, contributes to persistent lung inflammation and impaired alveolar development characteristic of bronchopulmonary dysplasia.

**What are the implications of the main findings?**

- The available evidence supports the involvement of IL-1β and IL-8 (CXCL8) in the inflammatory mechanisms associated with bronchopulmonary dysplasia in preterm infants.
- Further studies are needed to clarify the clinical utility of these cytokines as biomarkers and to determine whether modulation of these pathways may have therapeutic relevance in bronchopulmonary dysplasia.

**Abstract:**

**Background**: Bronchopulmonary dysplasia (BPD) is a leading chronic lung complication in extremely premature newborns. The etiological factors contributing to of BPD include both prenatal and postnatal risk factors, as well as activation of innate immunity. Innate immunity and its bioactive mediators play a central role in orchestrating the inflammatory response. Among these, interleukin-1β (IL-1 β) and IL-8 (CXCL8) are particularly prominent. **Methods**: A structured literature search was conducted across major biomedical databases (PubMed, Scopus, Web of Science, and Ovid MEDLINE) to identify relevant studies published between 1993 and November 2025. Article selection was guided by predefined inclusion criteria focusing on studies that examined IL-1β and IL-8 (CXCL8) in relation to bronchopulmonary dysplasia. Evidence from both human and animal studies was narratively synthesized. **Results**: This review provides a detailed description of the role of the innate immune system in BPD, including mechanisms of inflammatory initiation, evidence from human and animal studies on IL-1β and IL-8 (CXCL8), and the interaction between these two cytokines in the development of chronic lung disease. **Conclusions**: Both human and animal studies generally suggest that elevated levels of IL-1β and IL-8 (CXCL8) are closely associated with the development of bronchopulmonary dysplasia in premature infants.

## 1. Introduction

### 1.1. Epidemiology, Risk Factors, Pathogenesis and Genetics of Bronchopulmonary Disease

Bronchopulmonary dysplasia (BPD) is a chronic inflammatory lung disease affecting preterm newborns [1]. The most important risk factor for its development is low gestational age. Globally, an estimated 13.4 million neonates are born preterm each year [2]. In a systematic literature review, Siffel et al. (2021) reported wide variability in BPD incidence (10% to 89%) across Europe, North America, Asia, and Oceania, largely attributable to differences in diagnostic criteria, oxygen therapy practices, and preventive or therapeutic interventions [3]. In European studies, more than 80% of infants born at ≤24 weeks’ gestation developed BPD, compared with 8–38% of those born at ≥27 weeks [3]. Similar findings were reported in the USA, where 75% of infants born at 22–24 weeks and 49.8% of infants born at 22–28 weeks developed BPD [4].

In addition to low gestational age, several prenatal risk factors contribute to the development of BPD, including male sex, low birth weight, intrauterine growth restriction (IUGR), chorioamnionitis, maternal hypertension, and maternal smoking [5,6,7,8,9].

The pathogenesis of BPD involves both prenatal and postnatal activation of the innate and adaptive immune systems within the developing lungs in response to various injurious stimuli. Prenatally, immune activation may occur following fetal exposure to chorioamnionitis (CHA) [10].

The association between chorioamnionitis and BPD was first described by Watterberg et al. in 1996 [11]. Although subsequent studies produced inconsistent results [12], a large meta-analysis by Villamor-Martinez et al. confirmed chorioamnionitis as a significant risk factor for BPD, regardless of the diagnostic criteria used [13]. Supporting this observation, histological chorioamnionitis is present in nearly 40% of pregnancies ending before 28 weeks [10].

Colonization or infection with Ureaplasma species has also been identified as an important prenatal risk factor. In a study by Gobec et al., preterm infants colonized with *Ureaplasma* spp. had 4.32-fold increased odds of developing moderate-to-severe BPD [14].

Postnatally, lung injury is further exacerbated by invasive respiratory support, including endotracheal intubation, mechanical ventilation, and exposure to high concentrations of inspired oxygen. These factors, alone or in combination with late-onset sepsis, necrotizing enterocolitis (NEC), hemodynamically significant patent ductus arteriosus (hsPDA), and multiple blood transfusions promote persistent lung inflammation and fibrosis [7,8,9,15,16].

Genetic susceptibility to BPD has also been investigated. Early twin studies suggested a heritable component in the development of BPD [17]. However, a genome-wide association study (GWAS), conducted by Wang et al. failed to identify specific genetic loci or pathways accounting for BPD risk [18], highlighting the complex and multifactorial nature of the disease.

### 1.2. The Role of the Innate Immune System in the Development of BPD

Activation of the innate immune system plays a central pathogenetic role in the development of BPD in extremely premature newborns. Infectious and noninfectious stimuli activate alveolar macrophages, alveolar epithelial cells, pulmonary endothelial cells, dendritic cells, and resident innate lymphoid cells (ILCs), and attract other innate immune cells, triggering a cascade of inflammatory events that disrupt normal development of the alveoli and pulmonary vasculature.

### 1.3. Initiation of Inflammation (Figure 1)

The inflammatory process begins with lung tissue injury and subsequent activation of pulmonary endothelial and epithelial cells. Damaged epithelial and endothelial cells release damage-associated molecular patterns (DAMPs) and, in the presence of infection, pathogen-associated molecular patterns (PAMPs). These signals are recognized by pattern-recognition receptors (PRRs) expressed on immune cells and structural lung cells, including alveolar epithelial cells, such as Toll-like receptors (TLRs) and NOD-like receptors (NLRs) [19,20]. Engagement of PRRs by DAMPs and PAMPs activates downstream signaling pathways, most notably NF-κB activation and inflammasome formation, particularly the NLRP3 inflammasome, which drives the transcription of proinflammatory cytokines [21,22,23]. Activation of the NLRP3 inflammasome ultimately activates NF-κB, which is transcribed in the nucleus to express various proinflammatory cytokines and chemokines, including IL-1β and CXCL8 (IL-8) which play key roles in the development of chronic lung inflammation, such as that observed in BPD.

**Figure 1:**
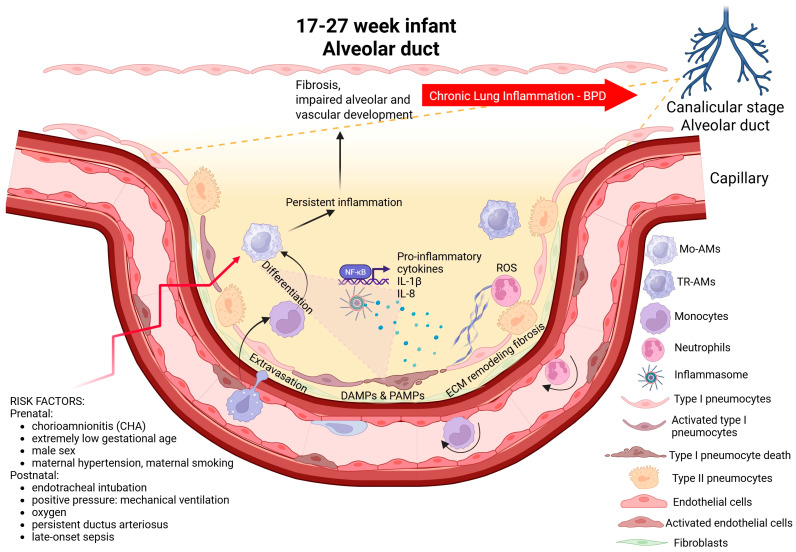
The role of innate immune system in development of BPD (TR-AMs, Tissue-Resident Alveolar Macrophages; MO-AMs, Monocyte-Derived Alveolar Macrophages; ECM, Extracellular Matrix; DAMP, Damage-Associated Molecular Pattern; PAMP, Pathogen-Associated Molecular Pattern; NF-κB; Nuclear Factor-Kappa B).

### 1.4. Cytokine and Chemokine Release

Upon inflammasome activation, epithelial, endothelial, and immune cells release potent pro-inflammatory mediators, including IL-1β, TNF-α, IL-6, and the chemokine IL-8 (CXCL8) [24,25]. These cytokines act synergistically to recruit and activate macrophages and neutrophils, amplifying the inflammatory response through the generation of reactive oxygen species (ROS), proteases, and additional pro-inflammatory cytokines. Persistent activation of the innate immune system results in sustained tissue injury, characterized by epithelial cell damage, fibroblast proliferation, and extracellular matrix remodeling, ultimately leading to impaired alveolarization and fibrosis—key pathological hallmarks of BPD [26].

### 1.5. Resolution of Inflammation

In tissues, inflammation is normally resolved through the action of anti-inflammatory cytokines, such as interleukin-10 (IL-10) and transforming growth factor- β (TGF-β), as well as specialized pro-resolving lipid mediators, including lipoxins and resolvins [27,28]. In premature infants, however, these regulatory pathways are immature, resulted in sustained activation of proinflammatory signaling and a diminished capacity for effective tissue repair. This imbalance promotes persistent lung inflammation and contributes to ongoing structural injury, thereby perpetuating lung injury [29].

In this article we present the results of a structured database search examining the role of IL-1β and CXCL8 (IL-8) in the pathogenesis of BPD.

## 2. Materials and Methods

### 2.1. Search Strategy

A structured electronic literature search was conducted across four major biomedical databases: PubMed, Scopus, Web of Science, and Ovid MEDLINE. The search aimed to identify relevant peer-reviewed articles published over a 32-year period, from 1 January 1993 to 30 November 2025. To ensure broad coverage of the available literature, search terms included combinations of Medical Subject Headings (MeSH), where applicable and free-text keywords, using Boolean operators (AND, OR). The core search strategy included terms related to “bronchopulmonary dysplasia”, “BPD”, “interleukin-1β”, “IL-1β”, “interleukin-8”, and “CXCL8”. To capture older literature, historical terminology for bronchopulmonary dysplasia, including “chronic lung disease”, “chronic lung disease of prematurity”, and “CLD”, was also incorporated into the search strategy.

Representative search strings were structured as follows: PubMed/Ovid MEDLINE: (“bronchopulmonary dysplasia”[Mesh] OR “bronchopulmonary dysplasia” OR “BPD” OR “Chronic Lung Disease of Prematurity”[Mesh] OR “chronic lung disease” OR “CLD”) AND (“interleukin-1beta”[Mesh] OR “IL-1beta” OR “IL-1β” OR “interleukin-8”[Mesh] OR “IL-8” OR “CXCL8”), Scopus/Web of Science: (TITLE-ABS-KEY(“bronchopulmonary dysplasia”) OR TITLE-ABS-KEY(“BPD”) OR TITLE-ABS-KEY(“chronic lung disease”) OR TITLE-ABS-KEY(“CLD”)) AND (TITLE-ABS-KEY(“IL-1beta”) OR TITLE-ABS-KEY(“IL-1β”) OR TITLE-ABS-KEY(“IL-8”) OR TITLE-ABS-KEY(“CXCL8”)). The search strategy was intended to capture relevant studies for narrative synthesis rather than to provide an exhaustive systematic retrieval.

### 2.2. Study Selection Criteria

Studies were selected for their relevance to the topic, with a focus on research investigating the role of IL-1β and IL-8 (CXCL8) in bronchopulmonary dysplasia. Particular emphasis was placed on studies involving neonatal populations, including preterm infants and animal models of neonatal lung injury, as well as studies reporting cytokine levels in biological samples such as bronchoalveolar lavage fluid, blood, serum, plasma, or tracheal aspirates. Priority was given to original research articles published in peer-reviewed journals. Review articles, meta-analyses, and relevant background literature were used to provide context but were not included in the primary evidence synthesis. Studies were excluded if they did not directly address the relationship between IL-1β and/or IL-8 and bronchopulmonary dysplasia, or if cytokine measurements were not reported.

### 2.3. Literature Selection Process

**Figure 2:**
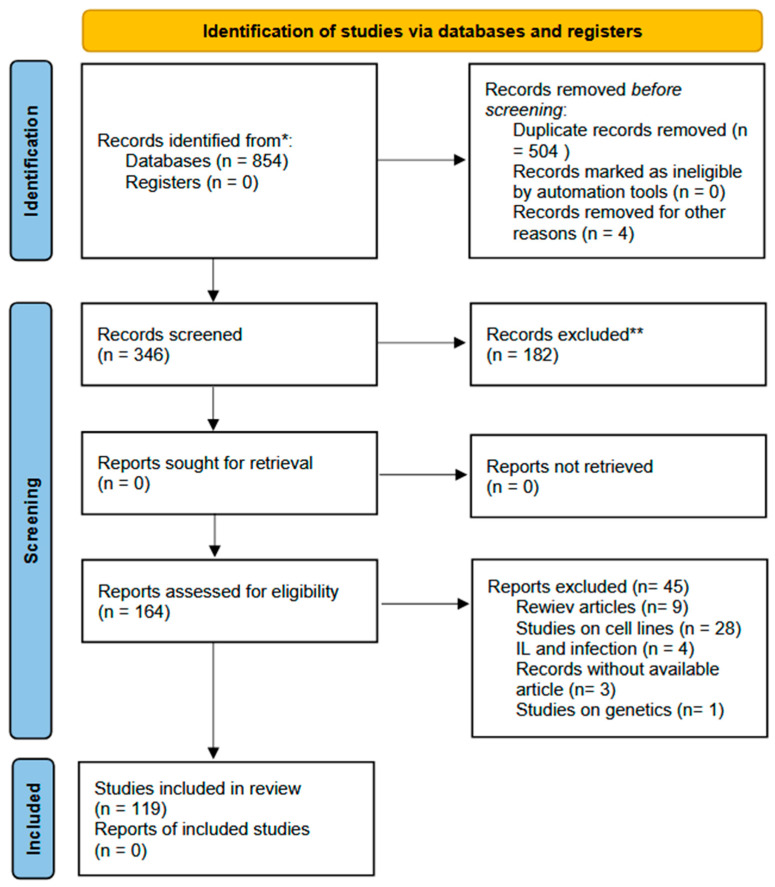
Overview of literature selection process. * Databases were searched to identify relevant records. ** Records were screened and excluded manually by the authors based on inclusion and exclusion criteria [30].

After the initial database search, duplicate records were removed and titles and abstracts were screened for relevance (Figure 2). Full-text articles were assessed as necessary to confirm their suitability for inclusion. Two reviewers (DBA and SG) independently conducted the selection process, resolving any disagreements through discussion and consensus. The final set of included studies was chosen based on relevance, methodological quality, and contribution to understanding the role of IL-1β and IL-8 in bronchopulmonary dysplasia.

Key characteristics of the included studies are summarized in Supplementary Table S1 (human studies) and Supplementary Table S2 (animal studies).

## 3. Cytokines

Cytokines are a broad group of low-molecular-weight peptides or glycoproteins produced by immune cells, primarily neutrophils, macrophages and T lymphocytes as well as structural lung cells, including epithelial and endothelial cells. They are broadly classified as interleukins, chemokines, interferons, and tumor necrosis factors and regulate immune responses through pro- and anti-inflammatory effects [31,32]. Cytokines act within a complex network and can exert antagonistic, additive, or synergistic effects [33].

### 3.1. IL-1β

Interleukin-1β (IL-1β), a member of the IL-1 cytokine family, plays a central role in immune-mediated and inflammatory diseases in both children and adults. It regulates cell proliferation, differentiation, and apoptosis and is critically involved in the initiation, maintenance, control and resolution of the inflammatory response [34].

### 3.2. Human Studies on IL-1β

Early studies demonstrated a link between chorioamnionitis, elevated IL-1β levels, and the development of BPD. Watterberg et al. first reported an association between histologically confirmed chorioamnionitis, or the presence of polymorphonuclear leukocytes in the amniotic fluid and the development of BPD in fifty-one mechanically ventilated premature infants. Lung inflammation was assessed in tracheal lavage fluid by measuring IL-1β, thromboxane B2, leukotriene B4, and prostaglandin E2. Chorioamnionitis was associated with early IL-1β elevation, reduced incidence of respiratory distress syndrome (RDS), and increased risk of BPD [11]. Similarly, Yoon et al. reported that elevated amniotic fluid concentrations of IL-6, TNF-α, IL-1β and IL-8 were significantly associated with BPD independent of gestational age [35]. Although some studies confirmed increased early postnatal cytokine levels following chorioamnionitis exposure [36], not all demonstrated a direct association with BPD incidence.

Elevated IL-1β levels in the early postnatal period have also been identified as a predictive marker of BPD. Increased concentrations of IL-1β, TNF-α and IL-6 in serum and tracheal aspirates within the first 24 h of life were associated with subsequent BPD development, although not with disease severity [37]. Similar associations between early elevation of inflammatory cytokines and BPD have been reported by other authors [38,39].

Longitudinal studies further highlighted the importance of sustained IL-1β activity. Rindfleisch et al. demonstrated a marked increase in IL-1β concentration and bioactivity during the first week of life in infants who developed BPD, accompanied by a relative imbalance between IL-1β and its endogenous antagonist IL-1Ra [40]. Although Kakkera et al. also observed increased levels of both IL-1β and IL-1Ra in ventilated preterm infants during the first week of life, they found no differences in the IL-1β/IL-1Ra ratio between BPD and non-BPD groups. This indicates that the rise in IL-1Ra is insufficient to counterbalance elevated IL-1β levels and prevent BPD development [41].

Broader cytokine profiling studies support these findings. Elevated levels of pro-inflammatory cytokines, including IL-1β, IL-6, IL-8, IL-10 and IFN-γ, along with lower concentrations of IL-17, RANTES, and TNF-β, were associated with BPD or death in extremely low birth weight infants [42]. Sahni et al. conducted an investigation across three neonatal intensive care units using scavenged blood from discarded samples. The infants were classified into five groups based on underlying pathology, and cytokine levels were measured at 36 weeks postmenstrual age (PMA). They found that, in infants with BPD-associated pulmonary hypertension (BPD-PH), higher levels of IL-1β may be associated with fibroblast stimulation and the development of pulmonary hypertension [43].

However, some studies reported that the associations between IL-1β and BPD were attenuated after adjustment for gestational age, highlighting its role as a major confounding factor [44].

Interventional and mechanistic studies have provided further insights. Less aggressive ventilation strategies, such as permissive hypercapnia, did not significantly reduce IL-1β levels or improve outcomes [45].

Tao et al. also reported noteworthy findings in a study conducted in two complementary directions; in vivo and in vitro. In the in vivo arm, the authors measured inflammatory cytokines levels in the sputum of premature infants born before 32 weeks of gestation. They found that during the first week of life, levels of all investigated pro-inflammatory cytokines were elevated in mechanically ventilated infants. However, by the fourth week of life, only IL-1β levels remained elevated, whereas the concentrations of the other interleukins had declined. The in vitro arm focused on the expression of miRNA-34a in airway epithelial cells and its relationship with IL-1β secretion. Using lentiviral vector, the researchers generated A549 cell lines with either overexpression or knockdown of microRNA-34a (miR-34a) and exposed them to hyperoxic conditions to mimic oxygen-induced lung injury. The results demonstrated that hyperoxia significantly increased the expression of miR-34a, which in turn positively regulated the pro-inflammatory cytokine IL-1β in a time- and concentration-dependent manner in A549 cells. Moreover, overexpression or knockdown of miR-34 would worsen or inhibit IL-1β production and its upstream signaling pathway of the NOD-, LRR-, and pyrin domain-containing protein 3 (NLRP3) inflammasome. Therefore, they are considered promising candidates for new targeted therapeutic approaches to lung diseases [46].

### 3.3. Animal Studies on IL-1beta

Animal studies have been instrumental in clarifying the role of IL-1β in the pathogenesis of BPD and in identifying both protective and injurious mechanisms affecting lung development. Most studies have been conducted in rodents due to their accessibility and short life cycles, although large-animal models have provided important translational insights. Transgenic and experimental models have demonstrated that IL-1β is sufficient to induce BPD-like lung injury. Conditional overexpression of IL-1β in lung epithelial cells results in pulmonary inflammation, impaired alveolarization, altered extracellular matrix deposition, and reduced vascular development, even in the absence of additional injury [47]. These models have also identified modulators of IL-1β-driven injury, including a protective role for matrix metalloproteinase-9 (MMP-9) [48] and a pathogenic role for β-6 integrin. In addition, they have shown that when maternal inflammation precedes fetal exposure, it may exert a protective effect on the neonatal lungs. In contrast, simultaneous inflammation in both the mother and fetus, abolishes this protective effect resulting in IL-1β -induced lung injury [49]. Furthermore, IL-1β-driven models have been used to investigate pulmonary retinoic acid signaling, developmental susceptibility and CXC chemokine receptor-2 (CXCR2)-mediated pathways in neonatal lung injury [50].

Pharmacological studies have confirmed the central role of IL-1 signaling. In a two-hit murine model combining antenatal inflammation and postnatal hyperoxia, treatment with an IL-1 receptor antagonist (IL-1RA) significantly reduced pulmonary inflammation and cytokine production, with protection dependent on the level of oxygen exposure [51]. Additional studies further highlighted the role of inflammasome-related pathways, showing that inhibition of caspase-8 or caspase-1 reduces IL-1β production, inflammatory cell recruitment, and lung injury [52,53,54]. Consistent findings across multiple models confirmed the importance of the NLRP3/IL-1β axis in hyperoxia- and infection-induced neonatal lung injury [55,56,57,58].

The study by Liao et al. clearly demonstrated the critical role of the NLRP3 inflammasome in the development of BPD. They exposed neonatal mice to hyperoxia which led to activation of the NLRP3 inflammasome, increased IL-1β levels, and reduced alveolarization. In contrast, Nlrp3−/− mice, which lack the NLRP3 gene, were protected from these effects, indicating the inflammasome’s essential role in BPD pathogenesis. Furthermore, pharmacological blockade of IL-1β using an IL-1 receptor antagonist (IL-1RA) or inhibition of NLRP3 inflammasome assembly with glyburide significantly reduced pulmonary inflammation and improved alveolarization in hyperoxia-exposed mice, suggesting potential therapeutic strategies for BPD. In addition to these experimental findings, the investigators demonstrated increased activation of the NLRP3 inflammasome, elevated IL-1β levels, and a higher IL-1β:IL-1RA ratio in both a ventilated preterm baboon model and samples from preterm infants. Importantly, these alterations correlated with increased pulmonary inflammation and impaired alveolar development and showed predictive value for the subsequent development of BPD [59]. Additional studies further highlighted the role of inflammasome-related pathways, showing that inhibition of caspase-8 or caspase-1 reduces IL-1β production, inflammatory cell recruitment, and lung injury [52,53,54].

Consistent findings across multiple models have confirmed the importance of the NLRP3/IL-1β axis in hyperoxia- and infection-induced neonatal lung injury [55,56,57,58].

Mechanical ventilation and oxygen toxicity are major postnatal drivers of IL-1β-mediated inflammation. While some studies in preterm lambs reported no significant differences in cytokine expression between ventilation strategies [60], others demonstrated that mechanical ventilation induces structural lung injury and increases IL-1β and pro-inflammatory gene expression [61]. Experimental studies further showed that ventilation strategies influence inflammatory responses, with optimized parameters, such as moderate positive end-expiratory pressure (PEEP), reducing cytokine expression and neutrophil activation [62]. Studies in utero and in early postnatal models of extremely preterm fetal sheep confirmed that even low tidal volume ventilation induces cytokine expression and structural changes in immature lungs [63]. These findings are further supported by additional studies linking mechanical ventilation to BPD-like lung injury [64].

Most experimental studies have focused on identifying substances that improve antioxidant status and reduce the synthesis of pro-inflammatory cytokines, particularly IL-1β and TNF-α. Protective effects against the development of BPD have been demonstrated for several categories of interventions, including endogenous proteins such as nesfatin-1, adiponectin and elafin [65,66,67], polyphenols [68,69,70,71,72,73], antioxidants and vitamins [74,75,76,77,78], prostaglandins [79], antibiotics [80,81], amino acids [82] and stem cell-based therapies [83,84,85,86,87,88].

Among these, vitamin D has shown particular promise, by attenuating hyperoxia-induced lung injury, reducing IL-1β levels, and improving vascular and alveolar development [89]. Additional studies have identified CCR5 as a key regulator of IL-1β-mediated inflammation, with CCR5 or IL-1β blockade improving lung structure in experimental BPD [90].

Zhang et al. established a neonatal model of BPD by administering intraperitoneal lipopolysaccharide (LPS) to pregnant rats. Using this model, they investigated the effects of rapamycin, an inhibitor of TOR phosphorylation involved in regulating cell pyroptosis. Their results demonstrated that rapamycin reduced activation of the NLRP3 inflammasome and significantly attenuated the degree of pyroptosis in BPD. These findings indicate that rapamycin promotes alveolar development in experimental BPD and suggest its potential therapeutic value [91].

### 3.4. IL-8/CXCL8

Interleukin-8 (IL-8), also known as CXCL8, is a potent CXC chemokine that plays a key role in inflammation by promoting leukocyte, particularly neutrophil, recruitment and activation at sites of tissue injury. Interleukin-8 was the first purified and molecularly cloned neutrophil chemoattractant identified from lipopolysaccharide-stimulated human mononuclear cells [92]. Its expression is tightly regulated at the transcriptional level, with NF-κB, AP-1, and C/EBP acting synergistically to induce IL-8 gene transcription in response to pro-inflammatory stimuli such as IL-1 and TNF-α [93]. IL-8 is synthesized as a precursor protein that undergoes proteolytic processing to generate multiple active isoforms, with shorter forms exhibiting greater neutrophil chemoattractant potency [93,94,95,96,97].

IL-8 exerts its biological effects through the G-protein-coupled receptors CXCR1 and CXCR2 [93]. It is an inducible cytokine produced by both immune and non-immune cells in response to inflammatory mediators such as IL-1 or TNF, microbial products, and environmental stressors such as hypoxia [98,99,100,101,102,103,104]. The biological effects of IL-8 are primarily reflected in its role in the coordinated recruitment of leukocytes to sites of inflammation. It is presented on the endothelial surface, where it promotes neutrophil adhesion by modulating integrin avidity, followed by transendothelial and transepithelial migration into inflamed tissues [105,106,107,108]. In addition to chemotaxis, IL-8 enhances neutrophil effector functions, including degranulation, oxidative burst, and the production of inflammatory mediators such as, leukotriene B4 and platelet-activating factor [109,110,111].

The role of IL-8 in neutrophil recruitment has been demonstrated in multiple experimental models. In rabbit models of acute inflammation, including glomerulonephritis and acute respiratory distress syndrome, neutralization of IL-8 significantly reduced neutrophil infiltration [112,113]. Similarly, in a mouse model of lipopolysaccharide-induced uveitis, blockade of the IL-8 receptor homologue reduced neutrophil infiltration without affecting rolling or adhesion, highlighting its specific role in leukocyte recruitment [114].

### 3.5. Human Studies

Human studies investigating IL-8 [CXCL8] in preterm infants have assessed cytokine levels in various biological compartments, including amniotic fluid, cord blood, tracheal aspirates, peripheral blood and lung tissue. These studies have also examined the influence of postnatal factors such as ventilation strategies, anti-inflammatory pharmacologic therapies, surfactant administration and nutritional support [115,116,117,118,119,120,121,122,123,124,125,126,127,128,129,130,131,132,133,134,135,136,137,138,139,140,141].

Early evidence indicates that elevated IL-8 levels are associated with an increased risk of BPD. Ghezzi et al. found that higher IL-8 concentrations in amniotic fluid predict BPD independently of gestational age and birth weight [115]. In this context, intra-amniotic infection—particularly with Ureaplasma species—has been strongly linked to elevated IL-8 concentrations [142,143,144,145,146,147].

Cord blood studies further support the role of IL-8 as a biomarker of disease severity. Rocha et al. analyzed venous cord blood concentrations of interleukins IL-1β, IL-6, IL-8, TNF-α and IL-10 in preterm infants born before 30 weeks of gestation and demonstrated that elevated IL-8 concentrations are associated with adverse outcomes, including death or moderate to severe BPD [148].

However, some studies suggest that this association may be attenuated after adjustment for gestational age, highlighting its role as a key confounder [149]. Nevertheless, IL-8, together with other inflammatory mediators, has been identified as an independent predictor of BPD in multivariate models [150]. Supporting these findings, De Dooy et al. demonstrated that elevated CXCL8 (IL-8) concentrations in tracheal aspirates obtained within the first two hours after birth were associated with prolonged respiratory support and adverse respiratory outcomes, particularly in extremely preterm infants born before 28 weeks of gestation [151].

IL-8 is a potent chemoattractant and one of the earliest chemokines to appear in the tracheobronchial tree in response to mechanical or oxidative stress, preceding leukocyte influx. In a study of 65 ventilated preterm infants (<32 weeks gestation) with RDS and no early infection, Munshi et al. found that IL-8 levels in tracheal aspirates were significantly elevated on postnatal days 1 and 3 in infants who later developed BPD, while neutrophil counts increased later (days 5 and 7). These findings indicate that early IL-8 elevation precedes neutrophil recruitment and supports its role as an initiating mediator of neutrophil-driven inflammation [152]. Furthermore, IL-1β has been shown to directly induce IL-8 expression in airway epithelial cells via NF-κB-dependent pathways, highlighting a functional link between these cytokines in ventilated preterm infants [153].

Different ventilation strategies and variations in tidal volumes used to ventilate premature infants have been extensively investigated in relation to BPD-like lung injury. Overall, these studies indicate that lung-protective, low-volume ventilation strategies are associated with lower airway concentrations of IL-8 and other pro-inflammatory cytokines, as well as a reduced risk of developing BPD [154,155,156,157].

In addition, anti-inflammatory therapies, including azithromycin and inhaled budesonide, significantly reduce IL-8 concentrations and improve respiratory outcomes, supporting the role of IL-8 as both a biomarker and a potential therapeutic target in BPD [158,159,160]. By contrast, immunohistochemical studies of autopsy lung tissue have yielded less consistent results. Although certain cytokines, such as IL-17, differ between BPD phenotypes, IL-8 expression has not consistently shown significant variation, and the lack of quantitative data limits interpretation [161].

Overall, human studies support a central role for IL-8 in lung inflammation, as it mediates neutrophil chemotaxis, regulates cytotoxic responses, and coordinates interactions among immune cells in preterm infants at risk of BPD.

### 3.6. Animal Studies

Investigating the influence of various factors on the regulation of inflammation through neutrophil activity has long been challenging, largely due to evolutionary differences between species. Rodents, the most commonly used experimental animals because of their small size, short life cycle and low maintenance cost, do not produce IL-8; instead, they express multiple functional homologues.

In mice, two IL-8 homologues interact with the CXCR2 receptor, whereas human IL-8 can activate both CXCR1 and CXCR2. These murine homologues share approximately 40–90% sequence homology with each other and with human IL-8 [93]. Studies by Coalson et al. in baboons, the phylogenetically closest animal, have enabled the incorporation of most prenatal (corticosteroids) and postnatal (surfactant, ventilation, nutrition, inflammation) factors involved in the development of BPD. This animal model has demonstrated clinical symptoms, histopathological changes, and inflammatory cell and interleukin profiles comparable to those in infants with BPD. Regarding interleukins, IL-8, unlike IL-1β, showed significantly increased levels in baboons, but only in association with lung inflammation, bronchopneumonia, atelectasis or patent ductus. In cases of sepsis without lung inflammation, IL-8 values were not elevated, while impaired alveolar and vascular development persists despite lung-protective ventilation strategies and minimal oxygen exposure [162].

In fetal sheep, Kramer and Jobe showed that intra-amniotic endotoxin exposure (chorioamnionitis model) increases IL-8 levels and induces lung inflammation, with endotoxin dose determining the timing and magnitude of the response [163]. Similar associations between amniotic fluid cytokines and BPD development were reported by McAdams and Cheah [164,165]. Antenatal endotoxin exposure further amplifies ventilation-induced inflammation in the preterm lung [166,167].

Wallace et al. showed that mechanical ventilation in immature lambs significantly increases pulmonary IL-6 and IL-8 mRNA expression compared with non-ventilated controls (*p* < 0.05) [168]. Similarly, Thompson demonstrated in a baboon BPD model that delayed extubation leads to elevated IL-6, IL-8, MCP-1, MIP-1α, and GRO-α levels, which are associated with increased neutrophil infiltration, likely driven by volutrauma and low-grade airway colonization [169].

Deng et al. showed that hyperoxia in neonatal rats increases CINC-1 and MIP-2 expression, promoting neutrophil accumulation and lung inflammation, while neutralizing antibodies reduce neutrophil influx, MPO activity, and preserve lung architecture [170]. Similar findings were reported by Vozeli and colleagues who demonstrated that anti-MCP-1 therapy attenuates neutrophil infiltration and protein oxidation, supporting a key role for chemokine-driven inflammation in hyperoxia-induced lung injury [171].

Animal models have also been used to evaluate the effectiveness of various therapeutic strategies in reducing IL-8 levels and attenuating inflammation in experimental settings. Corticosteroid–surfactant combinations improved oxidative lung injury in rabbits [172] and lambs [173], while mesenchymal stem cell-derived factors such as decorin showed protective effects [174]. In contrast, Kramer et al. reported in a sheep model that antenatal administration of retinoic acid, despite its well-known positive role in lung development, did not prevent structural lung changes induced by antenatal inflammation [175].

## 4. Discussion

This review provides a narrative synthesis of current evidence on the role of IL-1β and IL-8 (CXCL8) in the pathogenesis of bronchopulmonary dysplasia (BPD), highlighting a coordinated inflammatory axis that links early immune activation to sustained lung injury in preterm infants.

### 4.1. IL-1β as an Upstream Driver of Inflammation

IL-1β appears to play a central role in the inflammatory cascade in BPD. Clinical studies suggest that early elevation of IL-1β—often associated with chorioamnionitis and early postnatal inflammation—is predictive of subsequent BPD development [11,36,37,41]. Mechanistically, IL-1β production is thought to be driven by activation of the NLRP3 inflammasome, leading to downstream induction of additional pro-inflammatory mediators, including IL-6 and IL-8 [49,51,60,61]. Sustained IL-1β activity, particularly in the context of an imbalance with its endogenous antagonist IL-1Ra, may promote persistent inflammation and impaired lung development [40,41]. Experimental models further support this concept, showing that IL-1β overexpression induces BPD-like lung injury, whereas pharmacological blockade of IL-1 signaling attenuates inflammation and improves structural outcomes [49,51].

### 4.2. IL-8–Mediated Neutrophil Recruitment and Amplification of Injury

Downstream of IL-1β, IL-8 appears to function as a key effector of neutrophil-driven inflammation. Elevated IL-8 levels in amniotic fluid, cord blood, and tracheal aspirates have been associated with increased risk and severity of BPD [115,116,148,149,152,153]. Temporal studies suggest that IL-8 elevation precedes neutrophil influx, supporting its role as an initiating chemoattractant [153]. Once recruited, neutrophils may amplify lung injury through the release of reactive oxygen species, proteases, and additional cytokines, thereby perpetuating inflammation [105,106,107,109]. Importantly, broad-spectrum anti-inflammatory clinical interventions, such as azithromycin and inhaled budesonide, have been associated with improved respiratory outcomes alongside a secondary reduction in IL-8 levels. This suggests that while IL-8 may serve as a useful potential biomarker for monitoring treatment response, these drugs do not represent specific IL-8-targeted molecular therapeutics, highlighting a need for further targeted research [158,160].

### 4.3. Integration of Prenatal and Postnatal Inflammatory Signals

BPD is believed result from the convergence of prenatal and postnatal inflammatory exposures. Antenatal factors such as chorioamnionitis initiate cytokine activation [11,13], while postnatal insults—including mechanical ventilation, hyperoxia, and infection—amplify this response [60,61,62,63,64]. Animal models indicate that combined exposures cause in more severe lung injury than individual insults alone, underscoring the cumulative nature of inflammation in the developing lung [89,91,163]. Within this context, the IL-1β–IL-8 axis may represent a central mechanistic link between early immune activation and subsequent neutrophil-mediated injury. Mechanistically, early prenatal IL-1β elevation—often initiated by chorioamnionitis— has been shown to induce the transcription and expression of IL-8 via NF-κB in airway epithelial cells. This upstream activation may contribute to sustained inflammatory cascade that persists into the postnatal period, where it is further amplified by hyperoxia and mechanical ventilation, ultimately leading to extensive IL-8-driven neutrophil recruitment and structural lung damage. Understanding this temporal sequence may be important, as it suggests that therapeutic interventions targeting the IL-1β/NF-κB pathway could be most effective during the immediate perinatal window.

### 4.4. Cellular and Molecular Integration

The interaction between innate immune cells and structural lung cells plays a central role in orchestrating the inflammatory cascade in the developing lung. Alveolar macrophages function as primary sensors of tissue injury and release pro-inflammatory cytokines, including IL-1β and TNF-α, which subsequently activate epithelial and endothelial cells. In response, these cells produce IL-8 and other chemokines that drive neutrophil recruitment. Sustained neutrophilic inflammation results in excessive protease activity, including matrix metalloproteinase-9 (MMP-9) and neutrophil elastase, contributing to oxidative stress, and impaired alveolar and vascular development. In addition, emerging evidence points to regulatory mechanisms such as miR-34a-dependent activation of IL-1β via the NLRP3 inflammasome [46], suggesting complex, multifaceted control of innate immune activation in the developing lung.

### 4.5. Methodological Heterogeneity: BPD Definitions and Gestational Age as Confounders

While the association between the IL-1β–IL-8 axis and BPD is consistently reported in the literature, interpretation of these findings requires caution due to substantial methodological heterogeneity. A primary challenge lies in the evolving definition of BPD across historical cohorts. The included studies span more than three decades and apply varying diagnostic criteria, ranging from the classic Northway definition [1] to the more recent Jensen classification [176]. This shift in diagnostic criteria—from oxygen dependence at 28 days of life to graded severity based on respiratory support at 36 weeks postmenstrual age—affects patient classification and complicates direct comparisons of cytokine thresholds across studies. As highlighted in previous reports, the lack of diagnostic standardization contributes to wide variability in the reported incidence of BPD [3]. In addition, gestational age represents a major confounding factor [5,6], as extremely preterm infants exhibit both heightened inflammatory dysregulation and the highest incidence of BPD [4,9]. Consequently, it remains difficult to determine whether elevated IL-1β and IL-8 levels play a causal role or primarily reflect the immunological immaturity of the most premature infants [9,29]. Additional sources of variability include potential differences in baseline cytokine expression across different ethnic and geographical populations could not be fully assessed due to the predominance of heterogeneous, multicenter cohorts. Several methodological limitations of this review should also be acknowledged. As study selection and interpretation were not based on a fully systematic approach, the possibility of selection bias cannot be excluded. Furthermore, a formal risk-of-bias or quality assessment (e.g., Newcastle–Ottawa Scale or SYRCLE tool) was not applied, and the methodological quality of the included evidence could therefore not be quantitatively evaluated. In addition, this review was not prospectively registered. A critical clinical confounder across the included human cohorts is the variable rate of antenatal corticosteroid (ANS) exposure. As the standard of care for accelerating fetal lung maturity, prenatal corticosteroid administration is known to suppress inflammatory mediators and may therefore have influenced the baseline IL-1β and IL-8 expression profiles reported in some studies, further complicating comparisons of absolute cytokine concentrations across cohorts.

### 4.6. Translational Limitations of Animal Models Regarding IL-8 Biology

A key translational limitation arises when extrapolating mechanistic data from experimental models to human neonates, particularly regarding IL-8 biology. Rodent models (mice and rats), which constitute the vast majority of in vivo BPD research, lack a direct genetic ortholog for human IL-8 (CXCL8). Instead, rodent studies rely on functional equivalents, such as CXCL1 (KC) or CXCL2 (MIP-2), to model neutrophil chemoattraction [93]. While these murine chemokines signal through homologous CXCR1 and CXCR2 receptors [93], their regulation, temporal expression, and cellular sources differ from those of human IL-8. Larger animal models, such as preterm lambs or non-human primates, provide closer anatomical and physiological similarity and express IL-8 orthologs [162,163]. However, their use is limited by ethical, financial, and logistical constraints. Consequently, while rodent models have been indispensable for mapping the upstream role of IL-1β and general neutrophil influx, their applicability for validating targeted anti-IL-8 therapies remains restricted, highlighting the need for humanized models and clinical confirmation.

### 4.7. Clinical Implications

Overall, the available literature suggests that BPD is associated with a dysregulated balance between pro- and anti-inflammatory signaling within the innate immune system. Early perinatal inflammation and mechanical injury appear to initiate a self-sustaining cytokine network involving IL-1β, IL-6, and IL-8, which promotes neutrophil recruitment and contributes to alveolar injury and impaired repair. Modulation of innate immune pathways—including IL-1 blockade, NF-κB inhibition, macrolides, or corticosteroid-based approaches—has been explored as a potential strategy to reduce inflammation and improve outcomes in extremely preterm infants [51,158,160]. However, given the developmental context, therapeutic approaches must carefully balance suppression of harmful inflammation with preservation of essential immune and repair processes.

## 5. Conclusions

In conclusion, evidence from human and animal studies suggests an important involvement of IL-1β and IL-8 (CXCL8) in the pathogenesis of bronchopulmonary dysplasia. Acting within a coordinated inflammatory axis, IL-1β functions as an upstream regulator that initiates and sustains inflammatory signaling, while IL-8 is associated with neutrophil recruitment and amplification of tissue injury. The convergence of prenatal and postnatal inflammatory insults likely contributes to persistent activation of this pathway, ultimately impairing alveolar and vascular development in the immature lung.

These insights highlight the potential relevance of innate immune pathways in improving early risk stratification. However, given the developmental vulnerability of preterm infants, any therapeutic modulation of these pathways must carefully balance anti-inflammatory effects with the preservation of essential immune and repair mechanisms. Further well-designed studies are needed to clarify the precise role, clinical safety, and optimal timing of such interventions, as well as to identify reliable biomarkers for personalized approaches to BPD risk management.

Importantly, while the IL-1β–IL-8 axis has been consistently associated with the development and severity of bronchopulmonary dysplasia (BPD), a clear distinction must be maintained between causality and confounding. Elevated perinatal cytokine levels may partly reflect underlying immunological immaturity and overall illness severity of the lowest gestational age cohorts rather than acting solely as independent causal mediators. Future clinical studies and translational models should therefore account for key confounders, particularly gestational age in order to better define the independent pathogenic role of this inflammatory axis and to support the safe development of targeted therapeutic strategies.

## Data Availability

No new data were created or analyzed in this study.

## Supplementary Materials

The following supporting information can be downloaded at: https://www.mdpi.com/article/10.3390/children13070888/s1, Table S1: Summary of Human Studies Investigating IL-1β and/or IL-8 in Bronchopulmonary Dysplasia; Table S2: Characteristics and Main Findings of Animal Models Investigating IL-1β and IL-8 in Bronchopulmonary Dysplasia.

## Abbreviations

The following abbreviations are used in this manuscript:

BPD	Bronchopulmonary dysplasia
CHA	Chorioamnionitis
FIRS	Fetal inflammatory response
HA	Arterial hypertension
IUGR	Intrauterine growth restriction
CD	Cord blood
NEC	Necrotizing enterocolitis
HsPDA	Hemodynamically significant patent ductus arteriosus
TA	Tracheal aspirate
PRR	Pattern-recognition receptor
PAMP	Pathogen-associated molecular patter
DAMP	Damage-associated molecular pattern
NOD	Nucleotide-binding oligomerization domain
NLR	NOD like receptors
LRR	Leucin-rich repeat
NLRP3	NLR family pyrin domain containing 3
BALF	Bronchoalveolar lavage fluid
ELBW	Extremely low birth weight
IL	Interleukin
TLR	Toll-like receptors
CXCL	Chemokine
CXCR	Chemokine receptor
IFN	Interferon
