# Supplementary material for: Bronchopulmonary Dysplasia and Innate Immunity: A Narrative Review of the Roles of IL-1β and IL-8 (CXCL8)
Source: Children (Basel). 2026 Jul 1;13(7):888. doi: 10.3390/children13070888 (PMC13406551; doi:10.3390/children13070888)
Supplement: Supplementary file 1 [file children-13-00888-s001.zip › children-4355448-Supplementary Table S1.pdf]

Table S1. Summary of Human Studies Investigating IL-1β and/or IL-8 in Bronchopulmonary Dysplasia

Author, Year of publishing,	Title	Country	Number of patients, gestational age,	Biomarkers monitored, statistics (%, *P*)	Main Conclusions
**Human studies IL-1β**					
Watterberg et al., 1996. [11]	Chorioamnionitis and early lung inflammation in infants in whom bronchopulmonary dysplasia develops	Single country, 1 NICU (USA)	53 enroled, 41 survived; < 2000 gr<br><br>1,2,4 day of intubation	IL-1β, thromboxane B2, leukotriene B4, and prostaglandin E2 in tracheal lavages	Tracheal lavage concentrations of IL-1β were higher in infants in whom BPD developed.
Tullus et al., 1996. [38]	Elevated cytokine levels in tracheobronchial aspirate fluids from ventilator treated neonates with bronchopulmonary dysplasia	Single country, 1 NICU (Sweden)	Sample 1, TA, od 3 different categories, RDS, BPD, with/without steroids.	TNFα, IL-1β, IL-6, IL-8, IL-1ra	TNFα, IL-1β, IL-6, IL-8, and IL-lra were markedly elevated in tracheobronchial aspirates fluids from neonates with bronchopulmonary dysplasia. Corticoid treatment seemed to reduce these levels
Kotecha et al., 1996. [39]	Increase in Interleukin (IL)-1β and IL-6 in Bronchoalveolar Lavage Fluid Obtained from Infants with Chronic Lung Disease of Prematurity	Single country, 1 NICU (UK)	20 infants enrolled, 7 RDS, 9 CLD, 4 control BAL<br><br>Day 1, 4, 10, 17	IL-1β, IL-6, IL-8	IL-1β, IL-6, and IL-8 may contribute to the pathogenesis of CLD, and that, in CLD, IL-6 may be produced by cells within the air spaces.
Rindfleisch et al., 1996. [40]	Potential Role of Interleukin-1 in the Development of Bronchopulmonary Dysplasia	Single country, 2 NICU (USA)	30 patients, < 33 weeks, MV first day of life, 6 control infants TAL 1,3,5,7,14 and 28	IL-1 β, IL-1ra	Relative' imbalance of IL-1 β and IL-IRa may contribute to prolonged inflammation in BPD.
Yoon et al., 1997. [35]	Amniotic fluid cytokines (interleukin-6, TNF-α, IL-1-β, and interleukin-8) and the risk the development of bronchopulmonary dysplasia	Single country, 1 NICU (S.Korea)	Amniotic fluid	IL-6, TNFα, IL-1β, and IL-8	Antenatal exposure to proinflammatory cytokines is a risk factor for the development of bronchopulmonary dysplasia; the injury responsible for bronchopuimonary dysplasia in a subset of neonates may begin before birth.
Kakkera et al., 2005. [41]	Interleukin-1 balance in the lungs of preterm infants who develop	Single country, 1 NICU (USA)	35 enroled, GA< 30 weeks<br>TA 1,3,5 and 7 day	IL-1α, IL-1β IL-1ra;<br>$p < 0.05$	Infants not exposed to chorioamnionitis; levels of IL-1β and IL-1Ra, as well as neutrophils in the group of infants with BPD

	bronchopulmonary dysplasia				increased on days 5 and 7, compared to the 1st day of life.
Ambalavanan et al., 2009. [42]	Cytokines Associated with Bronchopulmonary Dysplasia or Death in Extremely Low Birth Weight Infants	Single country, 17 centers (USA)	1067 enroled, ELBW Infants: 401-1000 g Blood 4h,3,7,14, 21 days	IL-1β, IL-2, IL-8, TNF-α, RANTES, BDNF, CRP, GMCSF, IFN-γ, IL-4, IL-5, IL-6, IL-10, IL-12, IL-17, IL-18, MCP, MIP-1α, MIP-1β, MMP-9, NT4, sIL-6r, TGF-β, TREM1 Multi variable logistic regression	Higher serum concentrations of IL-1β, IL-6, IL-8, IL-10, IFN-γ and lower concentrations of IL-17, RANTES, and TNF-β were associated with the development of BPD/death in the extremely low birthweight (ELBW) infants
Stichel et al., 2011. [44]	Inflammatory cytokines in gastric fluid at birth and the development of bronchopulmonary dysplasia	Single country, 1 NICU (Sweden)	51 enroled, < 29 weeks Gastric fluid, within 1 h of birth	IL-1β, IL-8, Gro-α, ENA-78, CCSP<br><br>$p < 0.05$	Association between levels of proinflammatory cytokines, including IL-1β, exposure to chorioamnionitis, and the development of moderate and severe BPD. But, these associations were no longer significant after adjustment for gestational age.
Aghai et al., 2012. [36]	Impact of Histological Chorioamnionitis on Tracheal Aspirate Cytokines in Premature Infants	Single country, 1 NICU (USA)	40 enroled, GA < 30 weeks, BW > 1250 g TA samples, 48 hours after birth	IL-1α, IL-1β, and IL-8; $p \leq 0{,}02$	Infants who were exposed to chorioamnionitis had elevated levels of IL-1a, IL-1β, and IL-8.
Köksal et al., 2012. [37]	Value of serum and bronchoalveolar fluid lavage pro- and anti-inflammatory cytokine levels for predicting bronchopulmonary dysplasia in premature infants	Single country, 1 NICU (Turkey)	102 enroled, GA < 32 weeks Serum, TA First 24 hours of life	TNF-α, IL-1-β, IL-6, IL-10; $p<0.05$	Serum and tracheal aspirate concentrations of IL-1b, TNF-alpha and IL-6 at 24 hours of life were significantly higher in infants who developed BPD, compared to those who did not.
Gentner er al., 2017. [45]	Inflammatory Mediators in Tracheal aspirates of Preterm infants Participating in a randomized Trial of Permissive Hypercapnia	Single country, 1 NICU (Germany)	62 enroled, < 28 weeks, 401-1000 g TA from postnatal day 2–21.	IL-6, IL-8, IL-1β, IL-10, and MIP-1α; Mixed model two-way (factors being time and target group) analyses of variance (ANOVA) with a heterogeneous unstructured covariance	Higher target pCO2 values do not lead to reduced levels of IL-1β and other proinflammatory cytokines, and have no protective role

				structure SAS software 9.4	
Sahni et al., 2020. [43]	Novel biomarkers of bronchopulmonary dysplasia and bronchopulmonary dysplasia-associated pulmonary hypertension	Single country, 3 centers (USA)	90 patients Blood, At 36 weeks, 5 groups	IL -10, IL -13, IL - 1β, IL-6, IL-8, TNF- a, GM.CSF, VEGF, MCP-1, ICAM-1, CLIC 1, CLIC 4	ICAM-1 may be used as a specific biomarker for diagnosis of BPD and its severity.
Tao et al., 2022. [46]	Hyperoxia Induced Bronchopulmonary Dysplasia-Like Inflammation via miR34a-TNIP2-IL-1 Pathway	Single country, 1 NICU (China)	37 enroled, < 32 weeks; Human alveolar basal epithelial cells TA within first 24 hours, then every week during 5 weeks	IL-1β, TNF-α, Ang-1, COX-2 $p < 0.05$	In the first week, the levels of all tested proinflammatory cytokines were elevated in children on MV, but that by the fourth week of life, the levels of other interleukins fell, except for IL-1b, whose elevated value persisted
**Human studies IL-8**					
Groneck et al., 1994. [118]	Association of pulmonary inflammation and increased microvascular permeability during the development of bronchopulmonary dysplasia: a sequential analysis of inflammatory mediators in respiratory fluids of high-risk preterm neonates	Single country, 1 NICU (Germany)	24 patients, < 1200 gr<br><br>Tracheobronchial aspirates, 1st, 10th, 15th day	Complement component C5-derived anaphylatoxin, leukotriene B4, IL-8	Preterm neonates at risk for the development of BPD show an enhanced inflammatory reaction in the lungs and an associated increase in pulmonary microvascular permeability
Little et al., 1995. [124]	Role of elevated plasma soluble ICAM-1 and bronchial lavage fluid IL-8 levels as markers of chronic lung disease in premature infants	Single country, 1 NICU (UK)	17 infants, < 31 weeks GA, plasma and BAL, 1,5,14 DOL	ICAM-1, MPO, IL-8<br><br>Spearman's two tailed rank correlation. Mann-Whitney U test. T	The results indicate that high levels of plasma sICAM-1 and IL-8 in BLF at day 14 correlate with the development of chronic lung disease and indicate the severity of disease.
Jones et al., 1996. [127]	Undetectable Interleukin (IL)-10 and Persistent IL-8 Expression Early in Hyaline Membrane Disease: A Possible Developmental Basis for the Predisposition to	Single country, 1 NICU (USA)	terms (37-42 weeks) and preterms (23-32 weeks), 4 BAL samples during the first 96 hours of life,	IL-10, IL-1β, IL-8, TNF-α<br><br>Student t-test, 2 tal Fischer exact test	The study demonstrate that IL-10 mRNA and protein expression by lung inflammatory cells is related to gestational age, and during the first 96 h of life neutrophil cell counts and IL-8 expression decrease in BAL from term infants, but remain unchanged in

	Chronic Lung Inflammation in Preterm Newborns				BAL samples from preterm infants.
Munshi et al., 1997 [152]	Elevation of interleukin-8 and interleukin-6 precedes the influx of neutrophils in tracheal aspirates from preterm infants who develop bronchopulmonary dysplasia	Single country, 1 NICU (USA)	65 enroled, 56 completed, < 32 weeks<br><br>TA; Days 1, 3, 5, and 7	IL-8, IL-6; P<0,05	The level of IL-8 was statistically significantly higher in 1 and 3 days in the group of infants with BPD compared to those without BPD
Ghezzi et al., 1998 [115]	Elevated interleukin-8 concentrations in amniotic fluid of mothers whose neonates subsequently develop bronchopulmonary dysplasia	Single country, 1 NICU (USA)	47 enroled, 24-28 weeks<br><br>Amniotic fluid	IL-8	Sub-clinical intrauterine inflammation is a risk factor for the subsequent development of bronchopulmonary dysplasia. In utero aspiration of fluid with high concentration of pro-inflammatory mediators may contribute to the lung injury responsible for the development of bronchopulmonary dysplasia.
Takasaki et al. 1997 [122]	lnterleukin 8 and granulocyte elastase in the tracheobronchial aspirate of infants without respiratory distress syndrome or intrauterine infection and development of chronic lung disease	Single country, 1 NICU (Japan)	10 infants, < 1500 gr<br>TA 1-28 day, every 3 days	IL-8, E-α1 PI<br><br>Student's t-test, Cochran-Cox test and Chi-squared test<br>P = 0.05.	The present observations suggest that neutrophils, accumulated in the respiratory tract by the action of IL-8, release granulocyte elastase that causes tissue injury in this type of CLD.
Viscardi et al., 1997 [140]	Cromolyn Sodium Prophylaxis Inhibits Pulmonary Proinflammatory Cytokines in Infants at High Risk for Bronchopulmonary Dysplasia	Single country, 1 NICU (USA)	26 infants with high risk score, BAL, 3 and 7 DOL	TNF-a, sTNFR1, sTNFR2, IL-1b, IL-1ra, IL-8<br><br>Fisher's exact test, Student t test, Mann-Whitney U test or Wilcoxon rank sum test, Spearman Rank correlation P<0.05	Results suggest that nebulized DSCG may exert an anti-inflammatory effect in the lungs of infants < 1,000 g at risk for BPD
Jónsson et al., 1997 [126]	Early increase of TNFα and IL-6 in tracheobronchial aspirate fluid indicator of	Single country, 1 NICU (Sweden)	28 infants, < 34 weeks, intubated and MV, daily sample of TA	TNFα, IL-1β, IL-6, IL-8<br><br>Mann-	TA fluid cytokine concentrations may be used as a predictor of subsequent CLD and may help select a group of preterm infants

	subsequent chronic lung disease in preterm infants			Whitney U test, χ2 test, Spearman rank correlation coefficient test, p<0.05	at high risk of developing CLD for early treatment.
Niu et al., 1998 [120]	Early increase in endothelin-1 in tracheal aspirates of preterm infants: Correlation with bronchopulmonary dysplasia	Single country, 1 NICU (USA)	34 infants, 24-28 weeks GA, on days 1, 3, 5, and 7 of life	ET-1, IL-6, IL-8<br><br>Student's t test and the Fisher exact Test, nonparametric Mann-Whitney U test, Kruskal-Wallis ANOVA P<0.05	Early significant increase in the TA ET-1 and IL-8 concentrations in preterm infants with acute lung injury correlates with subsequent progression to BPD.
Thome et al., 1998 [154]	Comparison of Pulmonary Inflammatory Mediators in Preterm Infants Treated with Intermittent Positive Pressure Ventilation or High Frequency Oscillatory Ventilation	Single country, 1 NICU (Germany)	76 infants, 23/3-29/2 weeks, 420-1830 gr, TA, first 10 DOL	Albumin, IL-8, leukotriene B4	HFOV, when compared with a high rate low pressure IPPV, does not reduce concentrations of albumin, IL-8, and LTB4 in tracheal aspirates of preterm infants. requiring mechanical ventilation
Lyon et al., 1998 [141]	Randomised trial of erythromycin on the development of chronic lung disease in preterm infants	Single country, 1 NICU (UK)	75 infants < 30 weeks, BAL, 1-5 DOL	IL-1β and IL-8, TNF-α<br><br>Fisher's exact and Mann-Whitney U tests	U. urealyticum in the trachea was not associated with an increased inflammatory response in preterm infants. Erythromycin did not reduce the incidence or severity of CLD.
Gupta et al., 2000 [132]	Effects of Early Inhaled Beclomethasone Therapy on Tracheal Aspirate Inflammatory Mediators IL-8 and IL-1ra in Ventilated Preterm Infants at Risk for Bronchopulmonary Dysplasia	Single country, 2 NICU (USA)	161 infants, <1,251 g, <33 week, TA, 1,8,15,28 DOL	IL-8, IL-1ra<br><br>Nonparametric Wilcoxon rank sum test, Chi-square or Fisher exact tests, p<0.05	Early-inhaled beclomethasone therapy was associated with a reduction in pulmonary inflammation after 1 week of therapy.
Baier et al., 2001 [143]	Monocyte Chemoattractant Protein-1 and Interleukin-8 Are Increased in Bronchopulmonary Dysplasia: Relation	Single country, 1 NICU (USA)	35 infants, VLBW, TA, 1-2 DOL	IL-8, MCP-1<br><br>Student's t test, Wilcox rank sum test, p<0.05	Increased TA concentrations of IL-8 and MCP-1 during the first 2 weeks of life are associated with the development of BPD. Recovery of Uu from TAs is associated with a more robust inflammatory

	to Isolation of Ureaplasma Urealyticum				reaction and an increased risk of BPD.
D'Angio et al., 2002 [119]	Comparison of Tracheal Aspirate and Bronchoalveolar Lavage Specimens from Premature Infants	Single country, 1 NICU (USA)	40 infants, < 34 weeks, paired BAL and TA specimens were collected 1, 3, 7 and 28 days after birth	IL-8	Airway fluid levels of IL-8 are associated with the later development of BPD.
Shimotake et al., 2004 [153]	Interleukin (IL)-1 in Tracheal Aspirates from Premature Infants Induces Airway Epithelial Cell IL-8 Expression via an NF-B Dependent Pathway	Single country, 1 NICU (Japan)	18 enroled, <32 weeks<br><br>TA; the average day of life was 5.7 ± 1.1 d.	IL-8, IL-1 α, IL-1 β, TNF- α	Airway fluid from mechanically ventilated premature infants contains soluble factors capable of inducing airway epithelial cell IL-8<br>expression via a NF-B-dependent pathway, and that IL-1 plays a specific role in this process
An et al., 2004 [116]	Interleukin-6, interleukin-8, and soluble tumor necrosis factor receptor-I in the cord blood as predictors of chronic lung disease in premature infants	Single country, 2 NICU (Japan)	cord blood, 18 premature infants with CLD, 12 without CLD	IL-1β, IL-2, IL-4, IL-6, IL-8, IL-10, IFN-γ, TNF-α, soluble TNF receptor-I, soluble IL-6 receptor<br><br>Mann-Whitney test for continuous variables, $p < 0.05$	Elevated inflammatory cytokines in the cord blood are associated with the progression to CLD
Parikh et al., 2004. [135]	Effect of inhaled corticosteroids on markers of pulmonary inflammation and lung maturation in preterm infants with evolving chronic lung disease	Single country, 1 NICU (USA)	serum, preterm infants, 5 on beclomethason e therapy, 6 placebo, weight ≤ 1000 gr,	IL-8, TNF-α, IL-1α, sIL-2R	The addition of inhaled corticosteroids to a 7-day systemic course of corticosteroids did not alter cytokine response or improve pulmonary function.
Su et al., 2005 [123]	Interleukin-8 in bronchoalveolar lavage fluid of premature infants at risk of chronic lung disease	Single country, 1 NICU (Taiwan)	42 VLBW infants, BAL, 1-28 days, every 4 days	IL-8<br><br>$P<0.05$	Persistent inflammation could be a major contributory factor in the development of CLD.
Huang et al., 2005 [125]	Correlation of Augmented IL-8 Production to	Single country, 1 NICU (Taiwan)	34 premature infants, plasma and BAL, 1, 2, 4, and 7 DOL	IL-8, IL-10, TNF-α<br><br>Mann-Whitney U test, Wilcoxon signed ranks	Early pharmacologic regulation of IL-8 induction may be beneficial in the modulation of inflammatory reactions in premature infants.

	Premature Chronic Lung Disease: Implication of Posttranscriptional Regulation			test, Fisher exact test, P<0.05	
Gitto et al., 2005 [138]	Correlation among cytokines, bronchopulmonary dysplasia and modality of ventilation in preterm newborns: improvement with melatonin treatment	Single country, 1 NICU (Italy)	110 infants, < 32 weeks, serum, 1,3,7 DOL	IL-6, IL-8, TNF-α<br><br>Kolmogorov–Smirnov test, Wilcoxon rank test, Friedman and Kruskal–Wallis test. P<0.05	Melatonin treatment reduced the proinflammatory cytokines and improved the clinical outcome
Capoluongo et al., 2005 [156]	Comparison of serum levels of seven cytokines in premature newborns undergoing different ventilatory procedures: high frequence oscillatory ventilation or synchronized intermittent mandatory ventilation	Single country, 1 NICU (Italy)	40 infants, 24-29 weeks, Serum, 1,3,5 DOL	IL-6, IL-8, IL-10, MCP-1, PDGF-BB, VEGF and TGF-β1<br><br>two-tailed Fisher's exact test, both parametric and non-parametric tests, p<0.05	The results support the hypothesis that early use of HFOV, combined with an optimum volume strategy, has a beneficial effect, reducing serum levels of pro-inflammatory cytokines and consequently the acute phase leading to lung injury.
Bourbia et al., 2006 [121]	NF-kB in tracheal lavage fluid from intubated premature infants: association with inflammation, oxygen, and outcome	Single country, 1 NICU (USA)	33 infants, 24-31 weeks GA, TA sample collected on days 1–2, 3, 5, 7, and 14.	NF-kB, IL8<br><br>Unpaired t-test and rank sum tests, multiple logistic regression tests P<0.05	Tracheobronchial lavage NF-kB concentrations are related to lung inflammation, oxygen exposure, and pulmonary outcome in intubated preterm infants.
Dani et al., 2006 [155]	Effects of Pressure Support Ventilation Plus Volume Guarantee vs. High-Frequency Oscillatory Ventilation on Lung Inflammation in Preterm Infants	Single country, 1 NICU (Italy)	25 infants, < 30 weeks, BAL, samples were obtained from each patients within 30 min after the institution of HFOV or PSV + VG, and before surfactant treatment (T1), after 6–18 hr of ventilation (T2), after 24–	IL1b, IL-8, and IL-10<br><br>Student's t-test, Fisher's exact test, P < 0.05	Study demonstrates that early treatment with HFOV is associated with a reduction of lung inflammation in comparison with PSV þ VG in preterm infants with RDS.

			48 hr of ventilation (T3), and before extubation (T4)		
Lista et al., 2006 [157]	Lung Inflammation in Preterm Infants with Respiratory Distress Syndrome: Effects of Ventilation With Different Tidal Volumes	Single country, 1 NICU (Italy)	30 infants, 25-32 weeks, TA, 1, 3, 7 DOL	IL-6,IL-8, and TNF-α<br><br>Student's t-test, χ2 test, Mann-Whitney U-test, P<0.05.	Data show significantly higher lung inflammation in preterm infants ventilated with Vt¼3 ml/kg, suggesting a role for Vt¼5 ml/kg in reducing both inflammatory response during the acute phase of RDS and the length of ventilation.
De Dooy et al., 2007 [151]	High Levels of CXCL8 in Tracheal Aspirate Samples Taken at Birth Are Associated with Adverse Respiratory Outcome Only in Preterm Infants Younger than 28 Weeks Gestation	Single country, 1 NICU (Belgium)	141 enroled, 110 survived, < 28 weeks<br><br>TA; as soon as possible after birth but always within 2 hr	IL-1b, IL-6, IL-8, IL-10, IL-12p70 TNF- α<br><br>p <0.20	Only IL-8 showed an association with prolonged duration of ventilation but only in extreme premature infants
Aghai et al., 2007 [158]	Azithromycin Suppresses Activation of Nuclear Factor-kappa B and Synthesis of Pro-inflammatory Cytokines in Tracheal Aspirate Cells from Premature Infants	Single country, 1 NICU (USA)	10 enroled, < 28 weeks<br><br>One sample after intubation, TA	IL-6, IL-8<br><br>p< 0.05	Azithromycin in appropriate doses caused the suppression of NF-kappa B activation as well as the synthesis of proinflammatory cytokines IL-6 and IL-8 in a sample of TA cells in premature infants
Honda et al., 2009 [134]	Inhaled corticosteroid therapy reduces cytokine levels in sputum from very preterm infants with chronic lung disease	Single country, 1 NICU (Japan)	10 infants, < 28 weeks, < 1000 gr sputum	IL-8, TNF-α, IL-1β, IL-6, IL-10 , IL-12p70 Pearson's correlation coefficient, t-test, p<0.05	Inhaled corticosteroid therapy may be associated with a decrease in proinflammatory cytokine levels in sputum from infants with CLD from 2 weeks after the start of therapy
Paananen et al., 2009 [150]	Blood Cytokines during the Perinatal Period in Very Preterm Infants: Relationship of Inflammatory Response and Bronchopulmonary Dysplasia	Single country, 1 NICU (Finland)	128 infants, VLGA<br><br>Cord blood and plasma at 1 day and 7 days after birth	IL-6, IL-8, IL-10, and G-CSF	In infants exposed to CA, insufficient inhibition of high fetal inflammatory cytokine response shortly after birth may increase the risk of BPD
Kasper et al., 2010 [142]	The bacterial load of Ureaplasma parvum	Single country, 1	118 enroled, < 34 weeks	IL-8	U. parvum was the predominant biovar, and increased bacterial

	in amniotic fluid is correlated with an increased intrauterine inflammatory response	NICU (Austria)	Amniotic fluid		load was significantly linked to histologic chorioamnionitis, PROM + PL, early-onset sepsis, and bronchopulmonary dysplasia.
Yada et al., 2010 [146]	Association of development of chronic lung disease of newborns with neonatal colonization of Ureaplasma and cord blood interleukin-8 level	Single country, 1 NICU (Japan)	77 infants, < 32 weeks, cord blood	IL-8<br><br>Student's t-test, Mann–Whitney U-test, χ2 test, and logistic regression analysis	The development of CLD defined by oxygen requirement at 36 weeks was associated with neonatal Ureaplasma colonization but not with IL-8 level of cord blood.
Beeton et al., 2011 [145]	Role of pulmonary infection in the development of chronic lung disease of prematurity	Single country, 1 NICU (UK)	192 infants (term and preterm), gastric fluid, BAL, TA, NS aspirate, daily in first week, than twice weekly until 28 day	IL-6, IL-8<br><br>Mann–Whitney, Kruskal Wallis, Fisher's exact test<br>$P<0.05$	Both early and late microbial presence in neonatal lung fluid samples was significantly associated with the development of CLD suggesting that both ante- and post-natal infection play a role in the development of CLD.
Palojärvi et al., 2012 [131]	High tissue factor in lungs and plasma associates with respiratory morbidity in preterm infants	Single country, 1 NICU (Finland)	56 infants, <32 weeks, plasma and TA, 1,3, 7 DOL	TF, L-1β, IL-2, IL-4, IL-5, IL6, IL-7, IL-8, IL-10, IL-12, IL-13, IFN-c, GMSCFb, TNF-α<br><br>Mann–Whitney and Spearman correlation tests, $p<0.05$	Respiratory morbidity associates with high TF in lungs and plasma. In<br>sick newborn infants, upregulation of TF may be mediated by IL-6 and IL-8.
Rocha et al., 2012 [148]	Cord blood levels of IL-6, IL-8 and IL-10 may be early predictors of bronchopulmonary dysplasia in preterm newborns small for gestational age	Single country, 2 NICU (Portugal)	150 enroled, <30 weeks<br><br>Cord blood	IL-1β, IL-6, IL-8, IL-10, TNF-α<br><br>Chi-squared and the Mann-Whitney test.	IL-8 venous cord blood values were significantly elevated in patients who developed moderate/severe BPD when the outcome death or moderate/severe BPD was considered.
Chang et al., 2014 [137]	Mesenchymal Stem Cells for Bronchopulmonary Dysplasia: Phase 1 Dose-Escalation Clinical Trial	Single country, 1 NICU (South Korea)	9 infants, 23-29 weeks, 500-1250 gr, TA, 1,3,7 DOL	IL-6, IL-8, TNF-a, TGF-β, MMP-9	Intratracheal transplantation of allogeneic hUCB-derived MSCs in preterm infants is safe and feasible, and warrants a larger and controlled phase II study
Skouroliakou et al., 2016 [139]	Parenteral MCT/ω-3 Polyunsaturated Fatty Acid–Enriched	Single country, 1	60 infants, 26–32 weeks,	TNF–α, IL–6, IL-8, α-tocopherol, FAs	Compared with the soybean oil–based IVFE, the MCT/ω-3 PUFA-

	Intravenous Fat Emulsion Is Associated with Cytokine and Fatty Acid Profiles Consistent with Attenuated Inflammatory Response in Preterm Neonates: A Randomized, Double-Blind Clinical Trial	NICU (Greece)	serum, 1,15,30 DOL	MannWhitney U test, Fisher exact test P<0.05	enriched IVFE is associated with a more favorable cytokine and FA profile consistent with attenuated inflammatory response in preterm neonates.
Yilmaz et al., 2017 [117]	Low serum IGF-1 and increased cytokine levels in tracheal aspirate samples are associated with bronchopulmonary dysplasia	Single country, 1 NICU (Turkey)	40 enroled, <32 weeks<br><br>On postnatal day-1, 3, 7, 21 and 28 serum IGF-1 levels and IGF-1 levels, IL-6, IL-8, IL-10 and TNF-alpha levels in tracheal aspirate fluid samples of intubated cases were examined	IGF-1, IL-6, IL-8, IL-10, TNF-α<br><br>p<0.05	Levels of IL-6, IL-8, IL-10, and TNF-alpha in tracheal aspirate samples were significantly higher in cases with BPD compared to those without BPD
Leroy et al., 2018 [130]	A Time-Based Analysis of Inflammation in Infants at Risk of Bronchopulmonary Dysplasia	Single country, 1 NICU (Canada)	62 infants, <30 weeks GA, 1-42 DOL; serum	IL-6, IL-8, GCSF<br><br>P<0.05	Systemic inflammation occurs early in the neonatal period and precedes clinical symptoms in infants with BPD. These data provide a discrete vulnerability window period, supporting a role for targeted intensive care interventions during the early phase of BPD
Glaser et al., 2019 [147]	Perinatal Ureaplasma Exposure Is Associated With Increased Risk of Late Onset Sepsis and Imbalanced Inflammation in Preterm Infants and May Add to Lung Injury	Single country, 1 NICU (Poland)	103 infants, <1500 gr, < 32 weeks, cord blood	TNF-α, IL-1β, IL-8, IL-12, IL-17, IL10, IL-1ra, IFN-γ, IP-10, MMP-8/9, MIP-1α/β, MCP-1, VEGF, G-CSF, ICAM-1, VCAM-1,<br><br>P< 0.05	Positive Ureaplasma screening was not associated with BPD. However, exposure contributed to BPD in infants ventilated ≥5 days and conferred an increased risk of LOS and imbalanced inflammatory cytokine responses.
Nunes et al., 2020 [159]	Use of Azithromycin for the Prevention of Lung Injury in	Single country, 1	VLBW 40 + 40 patients	IL-1β, IL-2, IL-6, IL-8, and IL-10, TNF-α	Azithromycin has anti-inflammatory effects, with a decrease in cytokines after 5

	Mechanically Ventilated Preterm Neonates: A Randomized Controlled Trial	NICU (Brasil)	Blood, first and fifth day of life (before and 24 h after the last dose of azithromycin)		days of use and a reduction in death and O2 dependency at 28 days/death in mechanically ventilated preterm neonates.
Witkowski et al., 2020 [161]	Analysis of interleukins 6, 8, 10 and 17 in the lungs of premature neonates with bronchopulmonary dysplasia	Single country, 1 NICU (Brasil)	32 enroled, < 34 weeks<br><br>Pulmonary specimens	IL-6, IL-8, IL-10, IL- 17<br><br>$p < 0.05$	Only the level of IL-17 was statistically significantly different among the analyzed pathoanatomical samples (no BPD, new BPD, classic BPD), while the levels of IL-8 as well as the other tested cytokines did not differ
Zhang et al., 2021 [129]	Cytokines and Exhaled Nitric Oxide Are Risk Factors in Preterm Infants for Bronchopulmonary Dysplasia	Single country, 1 NICU (China)	46 infants, ≤32weeks days 1–3, 7–14, 21–28; venous blood	IFN-γ, IL-10, IL-12p70, IL-13, IL-1β, IL-2, IL-4, IL-6, IL-8,TNF-α t-test, Pearson's chi squared test or Fisher's exact test $p<0.05$	The cytokines may predict the occurrence of BPD to a certain extent.
Yao et al., 2021 [160]	Efficacy of budesonide in the prevention and treatment of bronchopulmonary dysplasia in premature infants and its effect on pulmonary function	Single country, 1 NICU (China)	94 enroled, < 32 weeks, < 1500 gr<br><br>Blood; Before and 4 weeks after treatment with BU	TNF-α, IL-6 and IL-8<br><br>$P <0.05$	Budenoside group showed statistically significantly higher decrease of inflammatory factors compared to control group.
Collaco et al., 2022 [128]	Perinatal Inflammatory Biomarkers and Respiratory Disease in Preterm Infants	Single country, 1 NICU (USA)	447 infants < 32 weeks, <1500 gr, plasma, first week of life	IL-6, IL-8, IL-10, VEGF<br><br>χ^2 tests, t tests, ANOVA; $p<0.05$	Preterm infants with higher levels of IL-6 and IL-8 during the first week of life were also more likely to be diagnosed with BPD.
Mehta et al., 2023 [136]	Extreme prematurity-associated alterations of pulmonary inflammatory mediators before and after surfactant administration	Single country, 1 NICU (USA)	40 infants, < 35 weeks, TA, 2 h after birth and 10-12 h after the administration of the first dose of surfactant.	IL-1β, IL-6, IL-8, IL-10, TNF-α, VEGF	Post-administration of surfactant, it is associated with a more prominent increase of VEGF, pro-inflammatory (IL-8, TNF- a) and antiinflammatory (IL-10) cytokines than in more mature preterm neonates.
Kayki et al., 2025 [149]	Cord blood chemokine levels as a predictor of oxidative stress and	Single country, 2 NICU (Turkey)	55 patients < 32 weeks<br><br>Cord blood	IL-8, IP-10, eotaxin, TARC, MCP-1, MIP-1α, MIG, ENA-78,	Cord blood chemokine levels including IL-8, MIP-3α, and MIP-1β may serve as potential biomarkers for identifying infants

	morbidities of prematurity			MIP-3α, GROα, I-TAC, MIP-1β	at risk of prematurity-related morbidities.
Li et al., 2025 [133]	Effect of Low Dose Glucocorticoid Inhalation on Bronchopulmonary Dysplasia in Premature Infants	Single country, 1 NICU (China)	144 infants, < 32 weeks, < 2500 gr, serum, before and 10 days after medication	IL-8, IL-10, TGF-β1<br><br>rank sum test and Chi square tests, $p<0.05$	Low-dose glucocorticoids have a signifi cant preventive and therapeutic effect on bronchopulmonary dysplasia in preterm infants, and have a high safety, showing high clinical application value for bronchopulmonary dysplasia in preterm infants.
