# Supplementary material for: Bronchopulmonary Dysplasia and Innate Immunity: A Narrative Review of the Roles of IL-1β and IL-8 (CXCL8)
Source: Children (Basel). 2026 Jul 1;13(7):888. doi: 10.3390/children13070888 (PMC13406551; doi:10.3390/children13070888)
Supplement: Supplementary file 1 [file children-13-00888-s001.zip › children-4355448-Supplementary Table S2.pdf]

Table S2. Characteristics and Main Findings of Animal Models Investigating IL-1β and IL-8 in Bronchopulmonary Dysplasia

Author, Year of publishing,	Title	Country	Animal model	Biomarkers monitored	Main Conclusions
**Animal studies IL-1β**					
Naik et al., 2001. [62]	Effects of Ventilation with Different Positive End-expiratory Pressures on Cytokine Expression in the Preterm Lamb Lung	1 laboratory (USA)	lambs	(IL)-1β, IL-6, IL-8, TNF-α	Study demonstrates that the initiation of mechanical ventilation in a preterm lung is injurious and that different ventilatory strategies influence the injury response.
Jobe et al., 2002. [60]	Decreased Indicators of Lung Injury with Continuous Positive Expiratory Pressure in Preterm Lambs	1 laboratory (USA)	lambs	IL-1, IL-6, IL-8	Lower levels of inflammatory molecules, as well as reduction of alveolar damage in the group of non-invasive ventilation vs. group on invasive ventilation.
Bry et al., 2006. [50]	Pathogenesis of Bronchopulmonary Dysplasia: The Role of Interleuken 1 in the Regulation of Inflammation-Mediated Pulmonary Retinoic Acid Pathways in Transgenic Mice	1 laboratory (Sweden)	mice	IL-1β	The study demonstrates a link between inflammation and the retinoic acid pathway. Inhibition of CRABP-I and RAR-2 expression may be one mechanism by which inflammation prevents alveolar septation.
Bry et al., 2007. [49]	IL-1 Disrupts Postnatal Lung Morphogenesis in the Mouse	2 laboratories (Sweden, USA)	mice	IL-1β	Perinatal expression of IL-1 in epithelial cells of the lung caused a lung disease that was clinically and histologically similar to BPD.
Lukkarinen et al., 2009. [48]	Matrix Metalloproteinase-9 Deficiency Worsens Lung Injury in a Model of Bronchopulmonary Dysplasia	1 laboratory (Sweden)	mice	IL-1β	IL-1b–induced lung injury was enhanced in infant transgenic mice lacking MMP-9 compared with mice with wild-type MMP-9 loci. Instead of playing a pathogenetic role in the development of BPD, MMP9 activity in the inflamed newborn lung may be a host-defense mechanism that protects

					the lung against inflammatory injury.
Hillman et al., 2010. [61]	Airway Injury from Initiating Ventilation in Preterm Sheep	2 centers (Australia, USA)	lambs	Egr-1, IL-1, MCP-1, IL-6	Egr-1, MCP-1, IL-6, and IL-1β mRNA increased in the lung tissue from fetal and newborn lambs on mechanical ventilation
Bry et al., 2010. [47]	Mechanisms of Inflammatory Lung Injury in the Neonate: Lessons from a Transgenic Mouse Model of Bronchopulmonary Dysplasia	1 laboratory (Sweden)	mice	IL-1β	Conditional expression of mature IL-1b in epithelial cells was related to inflammation, decreased vascular endothelial growth factor, abnormal a-smooth muscle actin and elastin deposition and disrupted alveolar septation
Brew et al. 2011. [64]	Injury and repair in the very immature lung following brief mechanical ventilation	1 laboratory, (Australia)	lambs	CTGF, EGR-1, CYR-61, IL-1β IL-6, IL-8, TNF-α, TGF-β	Brief MV of the very immature lung causes considerable structural injury in the parenchyma and bronchioles at 1 day. Immature lung is capable of virtually total repair within 15 days in the absence of further treatments.
Ozdemir et al., 2012. [73]	Colchicine Protects against Hyperoxic Lung Injury in Neonatal Rats	1 laboratory, (Turkey)	rats	MDA, TOS, GSH-Px, TNF-α, IL-1β,	Colchicine has favorable effects on alveolarization as well as inflammation and oxidative stress markers in an animal model of BPD.
Monz et al., 2013. [87]	Human Umbilical Cord Blood Mononuclear Cells in a Double-Hit Model of Bronchopulmonary Dysplasia in Neonatal Mice	1 laboratory (Germany)	mice	IL-1β, IL-2, IL-10, IL-6, TNFα, VEGF	Study demonstrate the therapeutic potential of umbilical cord blood MNCs in a new double-hit model of BPD in newborn mice. It was found improved lung structure and effects on molecular level.
Grisafi et al., 2013. [88]	Human Amniotic Fluid Stem Cells Protect Rat Lungs Exposed to Moderate Hyperoxia	1 laboratory (Italy)	rats	IL-6, IL-1b, IF-g, TGF-1b, VEGF	Treatment with hAFS cells has a reparative potential through active involvement of cells in alveolarization and angiogenesis.
Nold et al., 2013. [51]	Interleukin-1 receptor antagonist prevents murine	3 laboratories (Australia)	mice	IL-1β,	In mice treated with IL-Ra for 28 days, in contrast to treatment with vehicle, there

	bronchopulmonary dysplasia induced by perinatal inflammation and hyperoxia			IL-6, IL-1α, TREM-1, MIP-2, MIP 1α, TNF, KC, MIP-1β, BLC	was a decrease in inflammation and cytokine levels, but the response was only partial, depending on the degree of hyperoxia.
Liao et al., 2015. [59]	The NLRP3 inflammasome is critically involved in the development of bronchopulmonary dysplasia	1 laboratory (USA)	mice, baboons	IL-1β, IL1ra	Early activation of the NLRP3 inflammasome is a key mechanism in the development of BPD, and represents a novel therapeutic target for BPD
Xu et al., 2015. [72]	Resveratrol attenuates hyperoxia-induced oxidative stress, inflammation and fibrosis and suppresses Wnt/b-catenin signalling in lungs of neonatal rats	1 laboratory (China)	rats	MDA, TOS, GSH, TNF-α, IL-1β, IL-6	Resveratrol could protect lungs from hyperoxia-induced injury through its antioxidant, anti-inflammatory and anti-fibrotic effects.
Ozdemir et al., 2016. [77]	Dexpanthenol Therapy Reduces Lung Damage in a Hyperoxic Lung Injury in Neonatal Rats	1 laboratory, (Turkey)	rats	TNF-α, IL-1β	Dxp treatment results in less emphysematous change as well as decrease in inflammation and oxidative stress markers in an animal model of BPD.
Stouch et al., 2016. [56]	Interleukin-1β and Inflammasome Activity Link Inflammation to Abnormal Fetal Airway Development	4 laboratories (USA)	mice	IL-1β	In mouse lung explants, blocking IL-1β expression, post-translational processing, and signaling each protected the formation of new airways from the inhibitory effects of E. coli LPS
Hummler et al., 2017. [57]	Inhibition of Rac1 Signaling Downregulates Inflammasome Activation and Attenuates Lung Injury in Neonatal Rats Exposed to Hyperoxia	1 laboratory (USA)	rats	IL-1β,	Rac1 signaling regulates the expression of the inflammasome and plays a pivotal role in the pathogenesis of hyperoxia-induced neo natal lung injury.
Zhu et al., 2017 [85]	Human amnion cells reverse acute and chronic pulmonary damage in experimental neonatal lung injury	1 laboratory (Australia)	mice	TNF-α, IL-1β, MCP-1	Early hAEC treatment appears to be advantageous over late treatment. The benefits of hAEC administration resulted in long-term improvements in cardiorespiratory function.

Pan et al., 2018. [58]	Effects and molecular mechanisms of intrauterine infection/inflammation on lung development	1 laboratory (China)	rats	NLRP3, TNF-α, IL-1β, IL-6, VEGF, Collagen I, SP-A, SP-B, SP-C	NLRP3 inflammasome activation followed by inflammatory cytokines expression up-regulated, inhibiting the expression of pulmonary surfactant proteins, interfering with lung interstitial development. There are many identified miRNAs which target a wide range of genes and may play an important role in the processes of lung injury and BPD.
Dapaah-Siakwan et al., 2019. [54]	Caspase-1 Inhibition Attenuates Hyperoxia-Induced Lung and Brain Injury in Neonatal Mice	1 laboratory (USA)	mice	NLRP1, ASC, caspase-1, IL-1β and GSDMD	Hyperoxia activated the NLRP1 inflammasome, increased production of mature IL-1β and upregulated expression of p30 gasdermin-D (GSDMD), responsible for the programmed cell death mechanism of pyroptosis in both lung and brain tissue.
Ivanovska et al., 2020. [66]	Recombinant adiponectin protects the newborn rat lung from lipopolysaccharide-induced inflammatory injury	1 laboratory, (Canada)	rats	IL-1ß, MCP-1, MIP-1α	Recombinant APN blocks the deleterious pro-inflammatory effects of LPS on the immature lung whether it is given prior to or after the onset of inflammation.
Li et al., 2020. [67]	Recombinant Human Elafin Ameliorates Chronic Hyperoxia-Induced Lung Injury by Inhibiting Nuclear Factor-Kappa B Signaling in Neonatal Mice	1 laboratory (China)	mice	IL-1β, IL-6, IL-8, TNF-α	Elafin reduced apoptosis, suppressed inflammation cytokines, and improved NF-kB p65 nuclear accumulation in hyperoxia-exposed neonatal mice, indicating that this recombinant protein can serve as a novel target for the treatment of BPD.
Akduman et al., 2021. [76]	Astaxanthin Prevents Lung Injury Due to Hyperoxia and Inflammation	1 laboratory, (Turkey)	rats	GSH, TAS, TOS, LPO, 8-OHdG, AOPP, MPO, total thiol, TNF-α,	Astaxanthin was shown to reduce lung damage caused by inflammation and hyperoxia with its anti-inflammatory, anti-oxidant,

				IL-1β, caspase-3 activities	anti-apoptotic properties, and to protect the lung from severe destruction.
Ozdemir et al., 2021. [68]	Does Chrysin prevent severe lung damage in Hyperoxia-Induced lung injury Model?	1 laboratory, (Turkey)	rats	MDA, TOS, TNF-α, IL-1β	Prophylaxis with CH results in lower histopathological damage score and reduces apoptotic cell count, inflammation and oxidative stress while increasing anti-oxidant capacity.
Tayman et al., 2021. [75]	The therapeutic effect of Apocynin against hyperoxy and Inflammation-Induced lung injury	1 laboratory, (Turkey)	rats	TAS, TOS, OSI, AOPP, LPO, 8-OHdG, NADPH, NOX, SOD, CAT, GSH, MPO, TNF- α, IL-1β, IL-18, IL-6, caspase-1 and 3, NFR2, NLRP3	Apo was found to provide preventive and therapeutic effects by reducing oxidant stress, blocking inflammation and increasing antioxidant status. Also have anti-inflammatory effects by suppressing NLRP3 inflammasome activation and inducing Nrf2 as well.A
Chou et al., 2021. [86]	Consecutive daily administration of intratracheal surfactant and human umbilical cord-derived mesenchymal stem cells attenuates hyperoxia-induced lung injury in neonatal rats	1 laboratory (Taiwan)	rats	VEGF, PGDF, IL-1β, IL-6	Consecutive daily administration of intratracheal surfactant and hUC-MSCs can be an effective regimen for treating hyperoxia-induced lung injury in neonates.
Wang and Jiang., 2021. [89]	Role of vitamin D–vitamin D receptor signaling on hyperoxia induced bronchopulmonary dysplasia in neonatal rats	2 laboratories (China)	rats	IL-1β, IFN-γ, HIF-1α	Treatment with vitamin D3 resulted in a decrease of IL-1β and IFN-γ and an increase of HIF-1α in lung tissues under hyperoxia conditions.
Chen et al., 2021. [90]	CCR5 signaling promotes lipopolysaccharide induced macrophage recruitment and alveolar developmental arrest	1 laboratory (China)	rats	IL-1β, RIP3 antibody, phospho-RIP3 antibody, CCR5, Tublin phospho-p65 antibody,	Lipopolysaccharide-induced BPD rats have increased CCR5 and interleukin-1β (IL-1β) levels, and decreased alveolarization, while CCR5 or IL-1β receptor antagonist treatments decreased inflammation and increased alveolarization.

				CCL3, CCL4, CCL5	
Ozdemir et al., 2022. [71]	The protective effects of apocynin in hyperoxic lung injury in neonatal rats.	1 laboratory, (Turkey)	rats	MDA, TOS, TNF-α, IL-1β	This study reveal through an experimental neonatal hyperoxic lung injury that APO, an anti-inflammatory, antioxidant, and antiapoptotic drug, exhibits protective properties against the development of BP
Kryeziu et al., 2023. [69]	Quercetin supplementation attenuates airway hyperreactivity and restores airway relaxation in rat pups exposed to hyperoxia	1 laboratory (Kosovo)	rats	TNF-α, IL-1β	This study demonstrate the protective effect of quercetin on airway hyperreactivity and suggest that quercetin might serve as a novel therapy to prevent and treat neonatal hyperoxia-induced airway hyperreactivity and inflammation.
Wang et al., 2023. [74]	MSC-EXO and tempol ameliorate bronchopulmonary dysplasia in newborn rats by activating HIF-1α	1 laboratory (China)	rats	IL-1β, IL-17, IL-6, IFN-γ, HIF-1α, VEGF, p-PI3K, p-AKT	Combined treatment could improve lung tissue injury, promote pulmonary vascular remodeling, restore lung function, and inhibit oxidative stress in BPD rats. These effects were achieved through activation of HIF-1α.
Aslan et al., 2023 [78]	Molsidomine decreases hyperoxia-induced lung injury in neonatal rats	1 laboratory, (Turkey)	rats	TNF-α, IL-1β	Bronchopulmonary dysplasia may be prevented through the protective characteristics of MOL, an anti-inflammatory, anti-oxidant, and anti-apoptotic drug.
Chu et al., 2023. [81]	Erythromycin Attenuates Hyperoxia Induced Lung Injuryby Enhancing GSH Expression and Inhibiting Expressionof Inflammatory Cytokines	1 laboratory (China)	rats	GSH, TNF-α, IL-1β	Erythromycin may alleviate BPD by enhancing the expression of GSHand inhibiting the release of inflammatory mediators.
Ivanovski et al., 2023. [82]	L-citrulline attenuates lipopolysaccharide-induced inflammatory lung injury in neonatal rats	1 laboratory (Canada)	rats	IL-1β, IL-8, MCP-1α, TNF-α	The nonessential amino acid L-citrulline (L-CIT) mitigated lipopolysaccharide (LPS)-induced lung injury in the early stage of lung development in the newborn rat.

Zhang et al., 2023. [91]	Rapamycin attenuates pyroptosis by suppressing mTOR phosphorylation and promoting autophagy in LPS induced bronchopulmonary dysplasia	2 laboratories (China)	rats	Pro-caspase-1, caspase-1, NLRP3, Pro-IL-1β, IL-1β, IL-18/Pro-IL-18, GSDMD-N/GSDMD, Pro-caspase-11, caspase-11	Rapamycin reduces the activation of the NLRP3 inflammasome and attenuates pyroptosis levels in BPD
Azman et al., 2024. [63]	In utero ventilation induces lung parenchymal and vascular alterations in extremely preterm fetal sheep	1 laboratory, (Australia)	lambs	IL-1β, IL-6, IL-8, IL-10, TNF-α	24 hours of in utero ventilation, even at low tidal volumes, increased lung inflammation and surfactant protein expression and produced structural changes to the lung parenchyma and vasculature.
Reçica et al., 2024. [70]	Protective Effects of Resveratrol Against Airway Hyperreactivity, Oxidative Stress, and Lung Inflammation in a Rat Pup Model of Bronchopulmonary Dysplasia	1 laboratory, (Kosovo)	rats	TNF-α, IL-1β	This study demonstrate the protective effect of resveratrol against hyperoxia-induced airway hyperreactivity and lung damage and suggest that resveratrol might serve as a therapy to prevent the adverse effects of neonatal hyperoxia.
Sun et al., 2024. [80]	Adipose Stem Cells Derived Exosomes Alleviate Bronchopulmonary Dysplasia and Regulate Autophagy in Neonatal Rats	1 laboratory (China)	rats	TNF-α, IL-1β, IL-6, IL-10	The intratracheal administration of ADSC-Exos significantly improved alveolarization and pulmonary vascularization arrest in hyperoxia-induced BPD, which was associated with facilitating autophagy in part.
Chen et al., 2024. [84]	Adipose mesenchymal stem cells-derived exosomes attenuated hyperoxia-induced lung injury in neonatal rats via inhibiting the NF-κB signaling pathway	1 laboratory (China)	rats	TNF-α, IL-1β, IL-6, IL-10	AMSC-Exos attenuated the hyperoxia-induced lung injury in neonatal rats by inhibiting the NF-κB signaling pathway partly.
Huang et al., 2025. [52]	Caspase-8-driven NLRP3 inflammasome	1 laboratory (China)	mice	IL-1β, IL-18, caspase-1,	Selective targeted inhibition of caspase-8 reduces

	activation exacerbates bronchopulmonary dysplasia by increasing the apoptosis and pyroptosis crosstalk of alveolar epithelial cells			gasdermin-D (GSDMD), ASC	NLRP3 inflammasome activation, resists oxidative stress-induced lung injury, reduces the crosstalk between pyroptosis and apoptosis in lung epithelial cells, and reduces inflammatory immune cell infiltration and abnormal vascular remodeling.
León Silva et al., 2025. [53]	Caspase-1 inhibition mitigates neonatal hyperoxia-induced vascular and cardiopulmonary inflammation in neonatal rats	1 laboratory (USA)	rats	GSDMD, IL-1β, LOX, and TGF-β1, caspase-1	Inhibition of the caspase-1 pathway leads to decreased cardiopulmonary inflammation and remodeling. In conclusion, targeting caspase-1 signaling may be a therapeutic strategy to prevent the consequences of vascular and cardiopulmonary inflammation associated with preterm birth and oxygen therapy.
Tu et al., 2025. [55]	Macrophage pyroptosis mediates hyperoxia-induced inflammatory lung injury in neonates.	1 laboratory (China)	mice	NLRP3, Caspase-1 p20, NGSDMD, IL-1β	NLRP3/Caspase-1/GSDMD-mediated pyroptosis plays a critical role in hyperoxia-induced neonatal lung injury, and targeting this pathway may be beneficial for the prevention of lung injury in preterm infants
Yang et al., 2025. [65]	Nesfatin-1 regulates the H MGB1-TLR4-NF-κB signaling pathway to inhibit inflammation and its effects on the random skin flap survival in rats	1 laboratory (China)	rats	VEGF, IL-1β, TNF-α, HMGB1, TLR4, NF-κB p65	NES inhibited ischemic skin flap necrosis, promoted angiogenesis, and reduced ischemia-reperfusion injury and inflammation.
Yang et al., 2025. [79]	Effects of PGE1 on the ERS pathway in neonatal rats with hyperoxic lung injury	1 laboratory (China)	rats	IL-1β, IL-6, TNF-α, Caspase-3, CHOP, GRP78, Bcl-2/Bax protein expression	PGE1 treatment reduces levels of inflammatory cells and pro-inflammatory cytokines and decreases apoptosis. PGE1 has a therapeutic effect on BPD through the endoplasmic reticulum stress pathway.

Xu et al., 2025. [83]	Intratracheal administration of mesenchymal stem cells ameliorates hyperoxia-induced bronchopulmonary dysplasia by inhibiting NLRP3 inflammasome activation: the critical role of Aldh1a2	1 laboratory (China)	rats	IL-1β	The study revealed that UCMSCs mitigate lung injury by upregulating Aldh1a2, which suppresses NLRP3 inflammasome formation.
**Animal studies IL-8**					
Coalson et al., 1999. [162]	Neonatal Chronic Lung Disease in Extremely Immature Baboons	1 laboratory, USA	baboons	TNF-α, IL-6, IL-8, IL-1 β, IL-10	Model emonstrates that impaired alveolization and capillary development occur in immature lungs, even in the absence of marked hyperoxia and high ventilation settings
Deng et al., 2000. [170]	Lung Inflammation in Hyperoxia Can Be Prevented by Antichemokine Treatment in Newborn Rats	1 laboratory, USA	rats	CINC-1, MIP-2	Blocking neutrophilic chemokines with anti-CINC-1 and anti-MIP-2 antibodies effectively reduces neutrophil influx and prevents early hyperoxia-induced lung injury in newborn rats.
Kramer et al., 2001. [163]	Dose and Time Response after Intraamniotic Endotoxin in Preterm Lambs	2 laboratories, USA and Australia	lambs	IL-6, IL-8	Intraamniotic endotoxin induces fetal lung inflammation in a dose-dependent manner, and only higher doses that cause sufficient inflammation lead to lung maturation, while lower doses cause inflammation without improving lung function.
Yoder et al., 2003. [167]	Effects of Antenatal Colonization with Ureaplasma urealyticum on Pulmonary Disease in the Immature Baboon	1 laboratory, USA	baboons	IL-6, IL-8	Antenatal colonization with U. U. in immature baboons induces a persistent pulmonary inflammatory response that significantly accelerates and worsens the development of chronic lung injury and fibrosis.
Ikegami et al., 2004. [166]	Initial responses to ventilation of premature lambs exposed to intra-amniotic	1 laboratory, USA	lambs	IL-8, IL-1β, IL-6	Combining endotoxin-induced chorioamnionitis with gentle mechanical ventilation and surfactant

	endotoxin 4 days before delivery				treatment does not increase the acute inflammatory response in preterm lungs. This suggests a potential protective effect for the lungs under these specific, controlled conditions.
Vozzelli et al., 2004. [171]	Antimacrophage chemokine treatment prevents neutrophil and macrophage influx in hyperoxia-exposed newborn rat lung	1 laboratory, USA	rats	MCP-1, CINC-1	Blocking MCP-1 with neutralizing antibodies prevents the early influx of both macrophages and neutrophils into the lungs of hyperoxia-exposed newborn rats, reducing oxidative protein damage.
Thomson et al., 2006. [169]	Delayed Extubation to Nasal Continuous Positive Airway Pressure in the Immature Baboon Model of Bronchopulmonary Dysplasia: Lung Clinical and Pathological Findings	4 laboratories, USA	baboons	IL-6, IL-8 , MCP-1, Macrophage MIP-1α, GRO-α	Volutrauma and/or low-grade colonization of airways secondary to increased reintubations and ventilation times are speculated to play causative roles in the delayed nasal continuous positive airway pressure group findings.
Cheah et al., 2008. [164]	Oxidative Stress in Fetal Lambs Exposed to Intra-amniotic Endotoxin in a Chorioamnionitis Model	1 laboratory, Australia	lambs	IL-1β, IL-8	Fetal lambs exposed to intra-amniotic endotoxin in a chorioamnionitis model showed modest oxidative stress in the alveolar and systemic compartments, which was less extensive than the robust inflammatory response and early lung maturation, suggesting that oxidants are not major contributors to early lung maturation but may play a role in bronchopulmonary dysplasia.
Kramer et al., 2008. [175]	All-trans retinoic acid and intra-amniotic endotoxin mediated effects on fetal sheep lung	5 centers, Australia, Germany, USA, Nederland	lambs	IL-8	Fetal treatment with RA did not prevent inflammation-induced alveolar simplification

Wallace et al., 2009. [168]	Early biomarkers and potential mediators of ventilation-induced lung injury in very preterm lambs	3 laboratories, Australia	lambs	CTGF, CYR61, EGR1, IL1-β, IL-6, IL-8	Demonstrated that IL-6 and IL-8 mRNA levels were significantly higher in ventilated than in unventilated control lamb fetuses
Dani et al., 2011. [173]	Natural Surfactant Combined with Beclomethasone Decreases Lung Inflammation in the Preterm Lamb	1 laboratory, Italy	lambs	IL-6, IL-8, MIF	Combining natural surfactant with beclomethasone dipropionate at a dose of 800 μg/kg significantly reduces lung inflammation and oxidative stress while improving respiratory function in mechanically ventilated preterm lambs
McAdams et al. 2012. [165]	Choriodecidual Infection Downregulates Angiogenesis and Morphogenesis Pathways in Fetal Lungs from Macaca Nemestrina	1 laboratory, USA	monkeys	TNF-a, IL-8, IL-1β, IL-6	A transient choriodecidual infection may induce fetal lung injury with profound alterations in the genetic program of the fetal lung before signs of preterm labor.
Kwon et al., 2019. [174]	Decorin Secreted by Human Umbilical Cord Blood-Derived Mesenchymal Stem Cells Induces Macrophage Polarization via CD44 to Repair Hyperoxic Lung Injury	1 laboratory, South Korea	rats	IL-6, IL-8, IL-10, CINC-1	Decorin secreted by human umbilical cord blood-derived mesenchymal stem cells promotes the repair of hyperoxic lung injury by driving anti-inflammatory M2 macrophage polarization through direct interaction with the CD44 receptor
Gie et al., 2020. [172]	Intratracheal budesonide/surfactant attenuates hyperoxia-induced lung injury in preterm rabbits	1 laboratory, Taiwan	rabbits	IL-8, MCP-1, CTGF	Intratracheal administration of a budesonide/surfactant combination significantly improves lung compliance while reducing inflammation and structural remodeling in hyperoxia-exposed preterm rabbits.
